# Identification and characterization of novel filament-forming proteins in cyanobacteria

**DOI:** 10.1038/s41598-020-58726-9

**Published:** 2020-02-05

**Authors:** Benjamin L. Springstein, Christian Woehle, Julia Weissenbach, Andreas O. Helbig, Tal Dagan, Karina Stucken

**Affiliations:** 1 Institute of General Microbiology, Christian-Albrechts-Universität zu Kiel, Kiel, Germany; 2 Institute for Experimental Medicine, Christian-Albrechts-Universität zu Kiel, Kiel, Germany; 3 Department of Food Engineering, Universidad de La Serena, La Serena, Chile; 4 Present Address: Department of Microbiology, Blavatnick Institute, Harvard Medical School, Boston, MA USA; 5 Present Address: Max Planck Institute for Plant Breeding Research, Max Planck-Genome-centre Cologne, Cologne, Germany; 6 Present Address: Faculty of Biology, Technion-Israel Institute of Technology, Haifa, Israel

**Keywords:** Cellular microbiology, Bacterial genetics

## Abstract

Filament-forming proteins in bacteria function in stabilization and localization of proteinaceous complexes and replicons; hence they are instrumental for myriad cellular processes such as cell division and growth. Here we present two novel filament-forming proteins in cyanobacteria. Surveying cyanobacterial genomes for coiled-coil-rich proteins (CCRPs) that are predicted as putative filament-forming proteins, we observed a higher proportion of CCRPs in filamentous cyanobacteria in comparison to unicellular cyanobacteria. Using our predictions, we identified nine protein families with putative intermediate filament (IF) properties. Polymerization assays revealed four proteins that formed polymers *in vitro* and three proteins that formed polymers *in vivo*. Fm7001 from *Fischerella muscicola* PCC 7414 polymerized *in vitro* and formed filaments *in vivo* in several organisms. Additionally, we identified a tetratricopeptide repeat protein - All4981 - in *Anabaena* sp. PCC 7120 that polymerized into filaments *in vitro* and *in vivo*. All4981 interacts with known cytoskeletal proteins and is indispensable for *Anabaena* viability. Although it did not form filaments *in vitro*, Syc2039 from *Synechococcus elongatus* PCC 7942 assembled into filaments *in vivo* and a Δ*syc2039* mutant was characterized by an impaired cytokinesis. Our results expand the repertoire of known prokaryotic filament-forming CCRPs and demonstrate that cyanobacterial CCRPs are involved in cell morphology, motility, cytokinesis and colony integrity.

## Introduction

Species in the phylum Cyanobacteria present a wide morphological diversity, ranging from unicellular to multicellular organisms. Unicellular cyanobacteria of the *Synechocystis* and *Synechococcus* genera are characterized by a round or rod-shaped morphology, respectively, and many strains are motile. Species of the Nostocales order are multicellular and differentiate three types of specialized cells including heterocysts, which fix atmospheric nitrogen under aerobic conditions, hormogonia that are reproductive motile filaments and akinetes, which are dormant cells that are resistant to desiccation. Within the Nostocales, species of the Nostocaceae (e.g., *Anabaena*, *Nostoc*) form linear trichomes, while cells in the Hapalosiphonaceae and Chlorogloepsidaceae divide in more than one plane to form true-branching trichomes as in *Fischerella* or multiseriate trichomes (more than one filament in a row) as in *Chlorogloeopsis*^1^. Notably, cells within a single trichome of a multicellular cyanobacterium can differ in size, form or cell wall composition, which may be attributed to different stages of cell differentiation (or phenotypic heterogeneity) and varying environmental cues^2,3^. Cells in the *Anabaena* sp. PCC 7120 (hereafter *Anabaena*) trichome are linked by a shared peptidoglycan sheet and an outer membrane^4^. *Anabaena* cells communicate and exchange nutrients through intercellular cell-cell connections, called septal junctions, which are thought to comprise the septal junction proteins SepJ, FraC and FraD^5,6^. SepJ is essential for the multicellular phenotype in *Anabaena*^7,8^.

Studies of the molecular basis of cyanobacterial morphogenesis have so far focused on the function of FtsZ and MreB, the prokaryotic homologs of tubulin and actin, respectively^9^. FtsZ functions in a multi-protein complex called the divisome, and is known as a key regulator of cell division and septal peptidoglycan (PG) biogenesis^9,10^. FtsZ has been shown to be an essential cellular protein in *Anabaena* and in the coccoid cyanobacterium *Synechocystis* sp. PCC 6803 (hereafter *Synechocystis*)^11^. The FtsZ cellular concentration in *Anabaena* is tightly controlled by a so far undescribed protease^12^. Apart from its function in cell division, the FtsZ-driven divisome also mediates the localization of SepJ^13^. MreB functions in a multi-protein complex called the elongasome, where it is a key mediator of longitudinal PG biogenesis that controls the cell shape^9,14^. In cyanobacteria, MreB plays a role in cell shape determination in *Anabaena*, nonetheless, it is not essential for cell viability^15^. In contrast, in *Synechococcus* sp. PCC 7942 (hereafter *Synechococcus*) MreB is essential, where partially segregated mutants display a coccoid morphology resembling the morphology of *E. coli mreB* deletion strains^16,17^. In *Nostoc punctiforme* ATCC 29113, the MreBCD operon was shown to be regulated by the hormogonium-specific sigma factor SigJ and is likely involved in the transition of coccoid vegetative cells to the more rod-shaped cells that are characteristic to hormogonia^18^.

Proteins resembling the eukaryotic intermediate filaments (IFs) have been discovered in several bacterial species and were shown to form filaments *in vitro* and *in vivo* and to impact essential cellular processes^19^. IF proteins exhibit an intrinsic nucleotide-independent *in vitro* polymerization capability that is mediated by the high frequency of coiled-coil-rich regions in their amino acid sequence^9,20–22^. Eukaryotic IF proteins are generally characterized by a conserved domain buildup consisting of discontinuous coiled-coil segments that form a central rod domain. This rod domain is N- and C-terminally flanked by globular head and tail domains of variable length^22–24^. Crescentin is a bacterial IF-like CCRP from *Caulobacter crescentus*, which exhibits a striking domain similarity to eukaryotic IF proteins. Crescentin filaments that align at the inner cell curvature are essential for the typical crescent-like cell shape of *C. crescentus*; possibly, by locally exuding a constriction force which coordinates the MreB-driven peptidoglycan (PG) synthesis machinery^25–27^. Reminiscent of eukaryotic IF proteins, Crescentin was found to assemble into filamentous structures *in vitro* in a nucleotide-independent manner^25^. However, so far no Crescentin homologs have been found in other bacteria, indicating that non-spherical or rod-shaped prokaryotic morphologies are putatively controlled by other polymerizing proteins^28,29^. Apart from Crescentin, many other coiled-coil-rich proteins (CCRPs) with IF-like functions have been identified to polymerize into filamentous structures and to perform cytoskeletal-like roles; however, none of them resembled the eukaryotic IF domain architecture (reviewed by Lin & Thanbichler (2013)^19^). Examples are two proteins from *Streptomyces coelicolor* whose function has been studied in more detail: FilP and Scy^29–31^. Gradients of FilP localize at the tip of a growing hyphae and contribute to cellular stiffness^29^. Scy forms patchy clusters at the sites of novel tip-formation and, together with the scaffolding CCRP DivIVA, orchestrates the polar hyphal growth^30^. Together with FilP and a cellulose-synthase, these proteins form the polarisome, which guides peptidoglycan biogenesis and hyphal tip growth in *S. coelicolor*^30,32,33^. Another example are four CCRPs in the human pathogen *Helicobacter pylori*, which were found to assemble into filaments *in vitro* and *in vivo*, with a function in determination of the helical cell shape as well as cell motility^34,35^. Consequently, filament-forming CCRPs with essential cellular functions have been found in numerous prokaryotes having various cellular morphologies. The presence of filament-forming CCRPs in cyanobacteria is so far understudied. Here we search for CCRPs with presumed IF-like functions in cyanobacteria using a computational prediction of CCRPs. Putative filament-forming proteins were further investigated experimentally by structural analyses and *in vitro* and *in vivo* localization assays in morphologically diverse cyanobacteria.

## Results

### Coiled-coil-rich proteins are widespread in cyanobacteria

**Figure 1:**
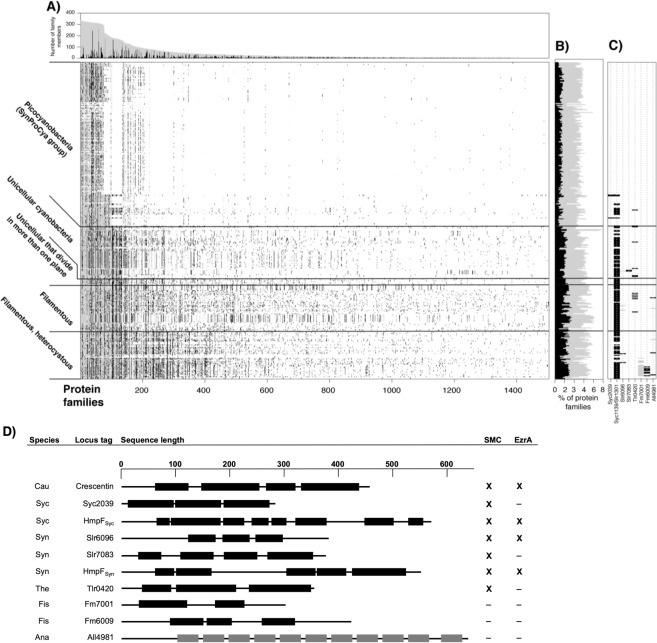
Distribution of CCRP protein families within cyanobacteria. (**A**) Lines in the presence/absence matrix designate cyanobacterial genomes; each column shows a protein family. Gray dots designate any homologous protein in the same protein family and black dots represent CCRP members. Protein families are sorted according to the number of members. Protein family size and the number of CCRP members are presented in a bar graph above. (**B**) The proportion of protein families containing CCRPs (gray) and CCRP proteins (black) in each genome. (**C**) Presence/absence pattern of CCRP candidate protein families. Only protein families with at least three members predicted to be CCRPs are shown. (**D**) Domain prediction of CCRP candidates. Scale on top is given in amino acid residues. Amino acid sequences in coiled-coil conformation are depicted by black bars with non-coiled-coil sequences represented by black lines. Tetratricopeptide repeats (TPR), also predicted by the COILS algorithm, are shown as grey bars. Proteins are given as cyanobase locus tags. Fm7001 and Fm6009 correspond to NCBI accession numbers WP_016868005.1 and WP_020476706, respectively. Abbreviations: Cau: *C. crescentus*; Syc: *Synechococcus*, Syn: *Synechocystis*; Ana: *Anabaena*; The: *Thermosynechococcus elongatus* BP-1; Fis: *Fischerella*. Cyanobacterial CCRPs had conserved domains present in prokaryotic IF-like CCRPs and eukaryotic IF proteins (Supplementary Table 1). Presence of a structural maintenance of chromosomes (SMC) domain or structural similarities to the cell division protein EzrA are marked with “**X**”, absence is indicated with “**−**”. Full list is given in Supplementary Table 1.

For the computational prediction of putative filament-forming proteins, we surveyed 364 cyanobacterial genomes including 1,225,314 protein-coding sequences (CDSs) for CCRPs. All CDSs in the cyanobacterial genomes where clustered by sequence similarity into families of homologous proteins (see Methods). The frequency of CCRPs in each CDS was calculated using the COILS algorithm^36^. The algorithm yielded a list of 28,737 CDSs with high coiled-coil content (≥80 amino acids in coiled-coil conformation; Supplementary File 1). CCRPs were predicted in 158,466 protein families covering all cyanobacterial species. To examine the overall distribution of CCRPs in cyanobacterial genomes, we investigated 1,504 families of homologous proteins that include at least three CCRP members (Fig. 1). Notably, most protein families (1,142; 76%) include CCRP and non-CCRP members, indicating that coiled-coil content may differ among homologous proteins. The presence/absence pattern of families including CCRPs further shows that those are less abundant in picocyanobacterial genomes (SynProCya group) in comparison to the remaining species in the phylum. Furthermore, the proportion of CCRPs in the genome is significantly higher in multicellular cyanobacteria in comparison to unicellular cyanobacteria (P = 2.65 × 10^−46^ using Kruskal-Wallis test and Tukey test with α = 0.05). This indicates that a high frequency of CCRPs is one characteristic of multicellular cyanobacteria.

For the experimental validation, the complete list of CCRPs was filtered to include candidates from freshwater unicellular and filamentous cyanobacteria that are genetically accessible, including *Thermosynechococcus elongatus* BP-1 (*Thermosynechococccus*), *Synechocystis*, *Synechococcus*, *Anabaena* and *Fischerella muscicola* PCC 7414 (*Fischerella*). In addition to cytoskeleton functions, coiled-coils are common motifs of proteins involved in other cellular processes such as transcription, the extracellular matrix, chemotaxis and host–pathogen interactions^37^. Consequently, the remaining CCPRs were further sorted to include proteins having similar properties to known prokaryotic IF-like CCRPs (e.g. Crescentin, FilP) and are annotated as hypothetical proteins with an unknown function. Additionally, proteins lacking an unstructured N-terminal head and C-terminal tail domain, which are characteristics of prokaryotic IF-like proteins^29^, were excluded. Furthermore, proteins with an assigned function or predicted to be involved in other cellular processes were excluded (using publicly available online bioinformatic tools: NCBI Blast, NCBI CD search, PSORTb, TMHMM, InterPro, PSIPRED and I-TASSER). In the screening for protein characteristics and annotation, Crescentin, FilP and other eukaryotic IF proteins (e.g., Vimentin and Desmin) were chosen as reference for our predictions, where proteins displaying similar results were favored. An additional *Fischerella* CDS, Fm7001, was added to the list as earlier analyses suggested that it has a cell shape-determining function. The preliminary filtration resulted in a list of nine candidates, which we investigated experimentally here (Fig. 1C,D and Supplementary Table 1).

Candidate coding sequences varied in size and ranged from ca. 280 amino acids (Synpcc7942_2039, abbreviated Syc2039) to ca. 650 amino acids (All4981). The coiled-coil domain distribution was variable among the candidates in both coiled-coil domain count and length (Fig. 1D). Only Slr7083 exhibited a somewhat characteristic domain architecture of eukaryotic IF proteins, whereas the coiled-coil domain distribution in the other candidates had major differences in coiled-coil domain number and lengths. None of the predicted CCRPs exhibited a stutter-like structure in the last coiled-coil segment. Besides coiled-coil domains, the COILS algorithm also predicted tetratricopeptide repeats (TPRs) as coiled-coils, thus we also included All4981 into our analysis, even though conserved domain searches reliably predicted these domains as TPRs and not coiled-coils. Many protein candidates contained conserved domains from eukaryotic IF proteins, found in Crescentin and FilP or from the bacterial cell division protein EzrA (Supplementary Table 1). The presence of these domains may be regarded as support for our classification. Additionally, structural maintenance of chromosomes (SMC) domains were predicted in almost all chosen candidates, all eukaryotic IF proteins as well as in Crescentin and FilP (Supplementary Table 1). The MscS_TM domain from Desmin was found in Slr7083 and Tlr0420 contains a Neuromodulin_N as well as a CCDC158 domain, both present in FilP or Crescentin, respectively.

The presence of homologs across all cyanobacterial morphotypes serves as a hint for universal protein function while a restricted distribution in specific subsections or morphotypes indicates a functional specialization within the respective taxon. An example for such species-specific candidate in our list is *slr7083* that is encoded on the pSYSA toxin-antitoxin plasmid in *Synechocystis*, similarly to *parM* and *tubZ*, which mediate plasmid segregation^38,39^. Synpcc7942_1139 and Slr1301 are homologs to the previously characterized motility protein HmpF from *N. punctiforme* hence we term them here HmpF_Syn_ and HmpF_Syc_, respectively, to highlight their relationship. In contrast to *slr7083*, the homologous proteins HmpF_Syn_ and HmpF_Syc_ are highly conserved and have homologs among all cyanobacterial groups but are absent from the picocyanobacterial (Fig. 1; Supplementary File 2). As most of the candidate CCRPs are annotated as hypothetical proteins, we initially verified the transcription of the respective genes by RT-PCR from cDNA (Supplementary Fig. 1A–D). Our results showed that *slr7083* was only weakly transcribed during mid-exponential culture growth phase and *all4981* was found to be transcribed in an operon with its upstream genes *all4982* and *all4983* (Supplementary Fig. 1B,C).

### Cyanobacterial CCRPs assemble into diverse filament-like structures *in vitro*

**Figure 2:**
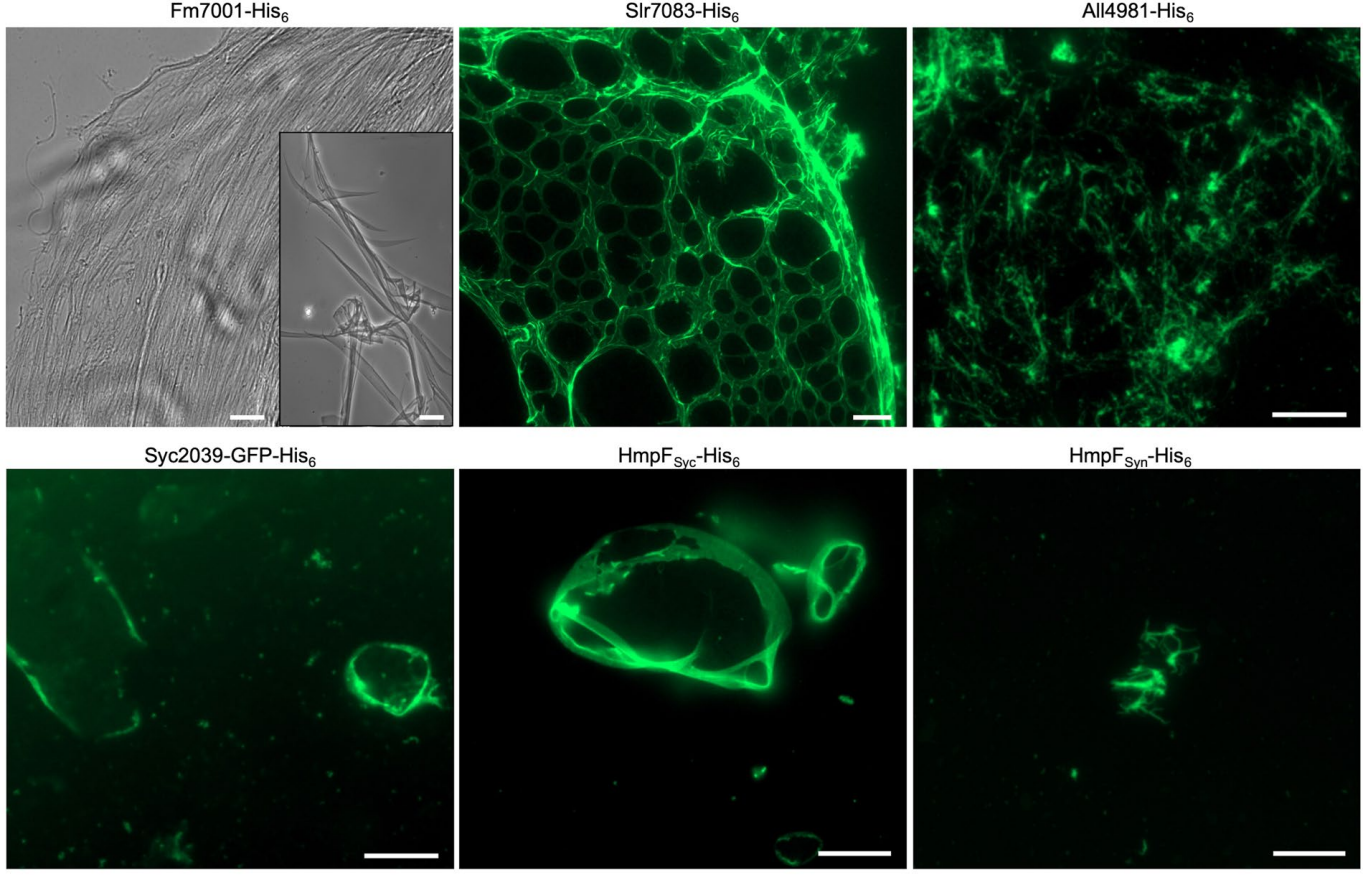
Cyanobacterial CCRPs assemble into diverse filament-like structures *in vitro*. Bright field and epifluorescence micrographs of filament-like structures formed by purified and renatured Fm7001-His_6_ (0.7 mg ml^−1^), Slr7083-His_6_ (1 mg ml^−1^), All4981-His_6_ (0.5 mg ml^−1^), Syc2039-GFP-His_6_ (0.3 mg ml^−1^), HmpF_Syc_-His_6_ (0.5 mg ml^−1^) and HmpF_Syn_-His_6_ (0.5 mg ml^−1^). Proteins were dialyzed into 2 mM Tris-HCl, 4.5 M urea pH 7.5 (Fm7001), HLB (Slr7083), PLB (All4981, HmpF_Syc_, HmpF_Syn_) or BG11 (Syc2039). Renatured proteins were either directly analyzed by bright field microscopy (Fm7001) or stained with an excess of NHS-Fluorescein and analyzed by epifluorescence microscopy. The NHS-Fluorescein dye binds primary amines and is thus incompatible with urea, which is why Fm7001 filament-like structures were visualized by bright field microscopy. Scale bars: 10 µm or (Fm7001 inlay and Slr7083) 20 µm.

A major characteristic of filament-forming proteins is their ability to self-polymerize into filaments intra and extracellularly^22,40^. Unlike actin and tubulin, IFs are able to form filamentous structures *in vitro* in a nucleotide-independent manner without additional co-factors upon renaturation from a denaturing buffer^40^. To examine the self-polymerization property of the nine tested CCRPs, we purified His_6_-tagged CCRPs under denaturing conditions and subjected them to subsequent renaturation by dialysis. Here we used protein concentrations in a similar range (0.5–1 mg ml^−1^) to previously investigated proteins shown to form filaments *in vitro* (e.g., Crescentin^25^ and Scc^41^, the metabolic enzyme CtpS^42^ and the bactofilins BacA, BacB^43^ and BacM^44^). When applicable, the purified proteins were labeled with NHS-Fluorescein and the formation of *in vitro* filaments was assessed by epifluorescence or bright field microscopy. Several candidates did not form discernible structures *in vitro* and were consequently excluded from further investigation (including Slr6096, Tlr0420 and Fm6009; Supplementary Fig. 2A). The remaining CCRPs assembled into highly diverse structures *in vitro* (Fig. 2). Direct dialysis of Fm7001 from a high urea-containing buffer to a physiological buffer led to protein precipitation. However, upon slow stepwise renaturation (removing 0.5 M every 2 h), Fm7001 polymerized into a flat two-dimensional sheet floating on top of the dialysate in 4,5 M urea (Supplementary Fig. 2D). We addressed the eventuality that these structures could be the product of crystalized urea, but control experiments did not reveal filaments. Polymerized Fm7001 revealed two-dimensional filament-like sheets as well as single filament-like fibers (Fig. 2). Similar structures were observed for purified Fm7001-GFP and MBP-Fm7001-His_6_ (Supplementary Fig. 2E,F). A two-dimensional filament-like pattern was observed also for Slr7083, which formed single, long and straight strings that were interconnected by two-dimensional sheets, thereby producing an irregular net (Fig. 2). Similarly, All4981 assembled into an interconnected net of thin and single filament-like strings (Fig. 2). The heterologous expression of Syc2039-His_6_ in *E. coli* failed, but we successfully purified Syc2039-GFP-His_6_ from *Synechococcus* instead. The polymerization pattern of Syc2039-GFP-His_6_ revealed sphere or cell shape-like three-dimensional sheets (Fig. 2). However, we note that most of the protein precipitated upon renaturation, hence it is unlikely that Syc2039 has *in vitro* polymerizing properties. HmpF_Syc_ polymerized into similar cell shape-like three-dimensional sheets but without any detectable aggregates (Fig. 2). The resemblance between Syc2039 and HmpF_Syc_ sheets raised the possibility that the sheet-like structures observed in the Syc2039-GFP-His_6_ sample represented co-precipitated and polymerized HmpF_Syc_. In accordance with this suggestion, we identified direct interactions of HmpF_Syc_ and Syc2039 using the bacterial adenylate cyclase two-hybrid (BACTH) assays (Supplementary Fig. 3A). For HmpF_Syn_, no clear *in vitro* structures were observed (Fig. 2). Notably, Crescentin, which we used as a positive control, polymerized into smooth and filigree filaments only in the presence of monovalent ions (i.e. NaCl; Supplementary Fig. 2B). This observation highlights the importance of suitable buffer conditions for the detection of filament-forming proteins. To further confirm our *in vitro* observations, we included the monomeric and highly soluble maltose binding protein (MBP) as well as the oligomeric proteins GroEL1.2^45^ (from *Chlorogloeopsis fritschii* PCC 6912) and the UMP kinase (from *Anabaena*) as negative controls. While both, the MBP and the UMP kinase readily clumped into comparably small aggregates, GroEL1.2 formed large proteinaceous aggregates *in vitro*, likely as a result of uncoordinated multimerization (Supplementary Fig. 2C). Consequently, we conclude that the *in vitro* filament-like structures of the cyanobacterial CCRPs we observed here are unlikely to be oligomerization artifacts. We further validated the self-binding properties of the remaining six CCRPs using the BACTH assay and found that all proteins are able to self-interact (Supplementary Fig. 3A).

### Putative filament-forming proteins form filament-like structures *in vivo*

To investigate whether the genetic background influences the polymerization properties of the candidate proteins, we expressed GFP or YFP translational fusion constructs of the putative filament-forming CCRPs in multiple hosts: (1) *E. coli*, (2) their native cyanobacterium and (3) in cyanobacteria of a different morphotype or subsection. Gene expression was driven by inducible or constitutive promoters commonly used in cyanobacteria. These included P_cpc560_ (for *Synechocystis*)^46^, P_trc_ (for *E. coli*, *Synechocystis* and *Synechococcus*)^47^ or P_petE_ (for *Anabaena* and *Fischerella*)^48^. As a positive control for *in vivo* polymerization, we expressed Crescentin-GFP in *Anabaena*, which formed round and helical filament-like structures in the cells, thereby showing that P_petE_ is suitable for studying filament-forming IF-like CCRPs in *Anabaena*. (Supplementary Fig. 4A).

### Fm7001 forms filament-like structures *in vivo* independent of the host

**Figure 3:**
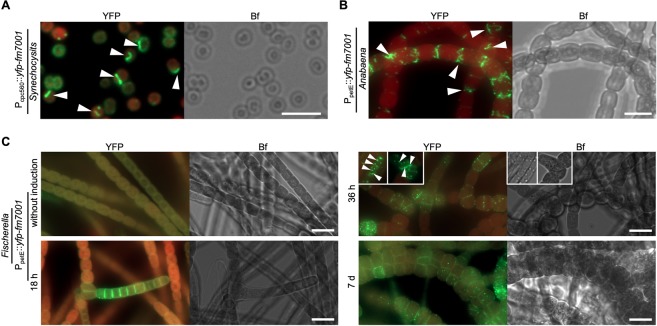
Host-independency for Fm7001 *in vivo* filamentation. Merged GFP fluorescence and chlorophyll autofluorescence (red) and bright field micrographs of (**A**) *Synechocystis*, (**B**) *Anabaena* or (**C**) *Fischerella* cells expressing YFP-Fm7001. Cells were either grown in (**A**,**B**) BG11 or (**C**) BG11 without copper and then induced with 0.5 µM CuSO_4_. (**C**) Micrographs were taken before induction of *yfp-fm7001* expression (without induction) and 18 h, 36 h or 7 d post induction. White triangles point to selected YFP-Fm7001 filament-like strings within the cells. Notably, unlike in *Anabaena* and *Fischerella*, Fm7001-GFP induced a swollen morphotype in *E. coli* and a subpopulation of *Synechocystis* cells (Supplementary Fig. 1E). (**B**) Maximum intensity projection of a Z-stack. Scale bars: **(A**,**B**) 5 µm, (**C**) 10 µm.

The *in vivo* localization of Fm7001 in *Fischerella* showed different results depending on the tag orientation. Only the expression of N-terminal YFP fusions of Fm7001 resulted in filament-like structures (Fig. 3 and Supplementary Fig. 4B). In *Synechocystis*, YFP-Fm7001 formed filament-like structures throughout the cell (Fig. 3A) while in *Anabaena* we observed septum-arising filament-like strings (Fig. 3B). In its host, *Fischerella*, YFP-Fm7001 only rarely assembled into short filament-like strings (Fig. 3C inlays). Despite of the low abundance of filament-like structures in *Fischerella*, induction of heterologous expression of YFP-Fm7001 induced an altered cell phenotype and trichomes seemingly divided in more than one plane resulting in a multiseriate (more than one trichome in a row) phenotype characteristic of *C. fritschii*. While under non-inducing conditions (i.e. in the absence of copper), *Fischerella* cells carrying a plasmid that expresses YFP-Fm7001 from P_petE_ had a WT phenotype, an altered morphotype and multiseriate growth was observed after around 4 rounds of replication (i.e. after 7 d) under inducing conditions (Fig. 3C). We also observed that, although expressed from a non-native promoter, YFP-Fm7001 was initially localized in the branching trichome, close to the branching points of putatively developing hormogonia (Fig. 3C, 18 h after induction). Those observations suggest that Fm7001 may be involved in cell shape control, branching phenotype or the development of hormogonia in *Fischerella*. Our attempts to generate a *Fischerella* ∆*fm7001* mutant strain remained unsuccessful, hence the function of Fm7001 remains unknown.

### Slr7083 and HmpF_Syn_ are involved in twitching motility in *Synechocystis*

**Figure 4:**
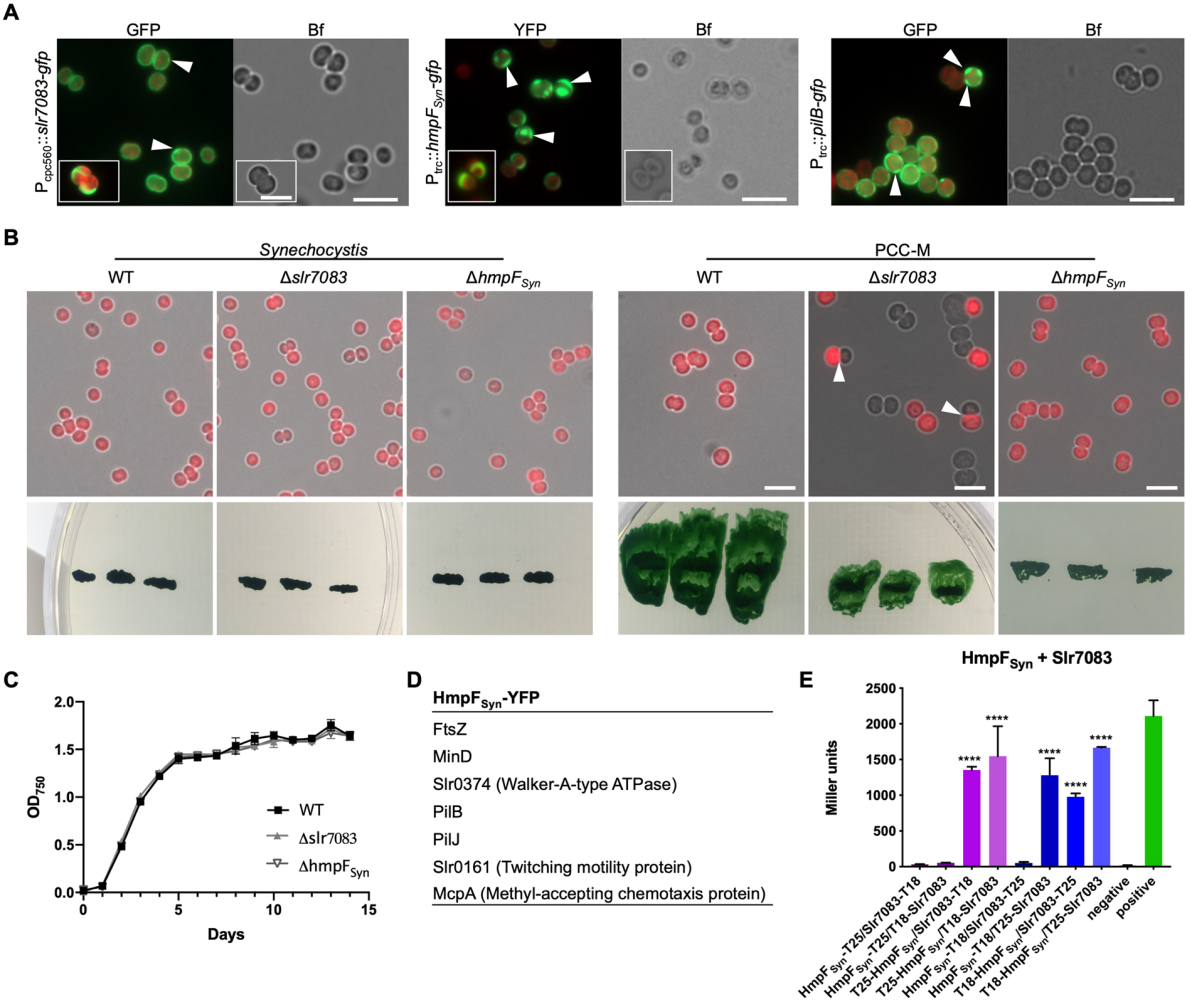
Slr7083 and HmpF_Syn_ are involved in twitching motility in *Synechocystis*. (**A**) Merged GFP fluorescence and chlorophyll autofluorescence (red) and bright field micrographs of *Synechocystis* cells expressing, Slr7083-GFP, HmpF_Syn_-YFP or PilB-GFP from P_cpc560_ (Slr7083) or P_trc_ (HmpF_Syn_, PilB). Expression of PilB-GFP in PCC-M resulted in the same localization pattern (data not shown). White triangles indicate focal spots and crescent-like formations. Scale bars: 5 μm. (**B**) Merged bright field and chlorophyll autofluorescence micrographs of motile and non-motile *Synechocystis* WT, Δ*slr7083* and Δ*hmpF*_*Syn*_ mutant cells. Below, motility tests of three single colonies from indicated cells streaked on BG11 plates and illuminated from only one direction are shown. (**C**) Growth curve of *Synechocystis* WT, Δ*slr7083* and Δ*hmpF*_*Syn*_ mutant strains grown in quadruples at standard growth conditions. OD_750_ values were recorded once a day for 15 d. Error bars show the standard deviation (n = 4). (**D**) Excerpt of interacting proteins of interest from mass spectrometry analysis of anti-GFP co-immunoprecipitations of *Synechocystis* cells expressing HmpF_Syn_-YFP from P_trc_. (**E**) Beta-galactosidase assays of *E. coli* cells co-expressing indicated translational fusion constructs of all possible pair-wise combinations of Slr7083 with HmpF_Syn_ grown for 1 d at 30 °C. Quantity values are given in Miller Units per milligram LacZ of the mean results from three independent colonies. Error bars indicate standard deviations (n = 3). Neg: pKNT25 plasmid carrying *hmpF*_*Syn*_ co-transformed with empty pUT18C. Pos: Zip/Zip control. Values indicated with * are significantly different from the negative control. *P < 0.05, **P < 0.01, ***P < 0.001, ****P < 0.0001 (Dunnett’s multiple comparison test and one-way ANOVA).

The *in vivo* localization of Slr7083-GFP in *Synechocystis* showed that it was localized to the cell periphery as well as rare focal spots spanning through the cell (Fig. 4A). We also attempted to localize Slr7083-GFP in the motile *Synechocystis* PCC-M substrain (hereafter PCC-M) but never obtained any successfully transformed clone, suggesting that overrepresentation of Slr7083 is deleterious for this strain. The localization of HmpF_Syn_-YFP in *Synechocystis* and PCC-M was at indistinct peripheral sites as assemblies of crescent-like shapes and rarely as focal spots spanning the cell (Fig. 4A and Supplementary Fig. 4C). Similar structures have been previously reported for the pilus ATPase PilB^49^. The localization of Slr7083-GFP and YFP-Slr7083 in *Anabaena* was at the cell periphery (Supplementary Fig. 4D). Furthermore, extended expression of YFP-Slr7083 in *Anabaena* altered the cellular morphology and disturbed the linear *Anabaena* trichome growth pattern (Supplementary Fig. 4E). In *E. coli*, Slr7083-GFP localized next to the cell poles (Supplementary Fig. 1E). When expressed in *E. coli*, HmpF_Syn_-GFP revealed a similar polar localization (Supplementary Fig. 1E). To further assess the role of HmpF_Syn_ and Slr7083 in *Synechocystis* motility, we generated *Synechocystis* and PCC-M Δ*slr7083* and Δ*hmpF*_*Syn*_ mutant strains. The *Synechocystis* Δ*slr7083* and Δ*hmpF*_*Syn*_ mutants revealed no phenotypic defects compared to the WT (Fig. 4B,C). In contrast, the PCC-M Δ*slr7083* mutant is characterized by a decrease in twitching motility and a defect in cytokinesis (Fig. 4B). PCC-M Δ*slr7083* mutant cells often lacked internal chlorophyll signal entirely and failed to properly divide internal thylakoid membrane (assessed by the lack of chlorophyll autofluorescence) during cell division (Fig. 4B). Similarly, the PCC-M Δ*hmpF*_*Syn*_ mutant lost its twitching motility (Fig. 4B) confirming previous results in *Synechocystis*^50^ and as also shown for *N. punctiforme* Δ*hmpF* mutants^51^. Attempts to complement the motility defect in the PCC-M Δ*hmpF*_*Syn*_ mutant by expressing HmpF_Syn_-YFP from the conjugation plasmid pRL153 failed, possibly as a result of the comparably high expression of HmpF_Syn_-YFP from P_trc_ (we note that P_trc_ cannot be regulated in *Synechocystis*). Alternatively, the addition of a fluorescent protein to the C-terminus of HmpF_Syn_ could have also rendered it non-functional. Notably, a C-terminal GFP fusion to HmpF from *N. punctiforme* was shown to be functional^51^, suggesting that HmpF_Syn-_YFP is likely also functional but complementation is prevented due to its overexpression. Additional attempts to complement the PCC-M Δ*slr7083* mutant never resulted in exconjugants. In order to further explore how HmpF_Syn_ affects motility, we analyzed co-precipitated proteins of HmpF_Syn_-YFP expressed in *Synechocystis* by mass spectrometry. This revealed multiple putative interaction partners involved in motility, including a twitching motility protein (Slr0161), two methyl-accepting chemotaxis proteins (McpA and PilJ) and the type IV pilus assembly ATPase PilB (Fig. 4D). The interaction of HmpF_Syn_ with PilB, together with their similar *in vivo* localization, prompted us to characterize the interaction of both proteins. For this purpose, we attempted to express PilB-GFP in *Synechocystis* WT, and in the Δ*hmpF*_*Syn*_ and Δ*slr7083* mutants. In *Synechocystis* WT, PilB-GFP localized to the cell periphery and often formed crescent-like formations (reminiscent of HmpF_Syn_-YFP and Slr7083-GFP; Fig. 4A), confirming previous results^49^. However, we never observed any PilB-GFP expression in the *Synechocystis* or PCC-M Δ*slr7083* and Δ*hmpF*_*Syn*_ mutants (Supplementary Fig. 5) and the few obtained exconjugants were never viable upon re-streaking on fresh selective plates or transfer to liquid growth medium. The similarity between our observations so far for HmpF_Syn_ and Slr7083 led us to test for an interaction between these two proteins. Indeed, a bacterial two-hybrid assay confirmed a direct interaction between Slr7083 and HmpF_Syn_ (Fig. 4E). Taken together, our investigation identified two *Synechocystis* CCRPs that are involved in cell motility and are localized to the cell periphery, often as crescent-like structures.

### All4981 is an *Anabaena* TPR protein that forms septal-arising filament-like structures

**Figure 5:**
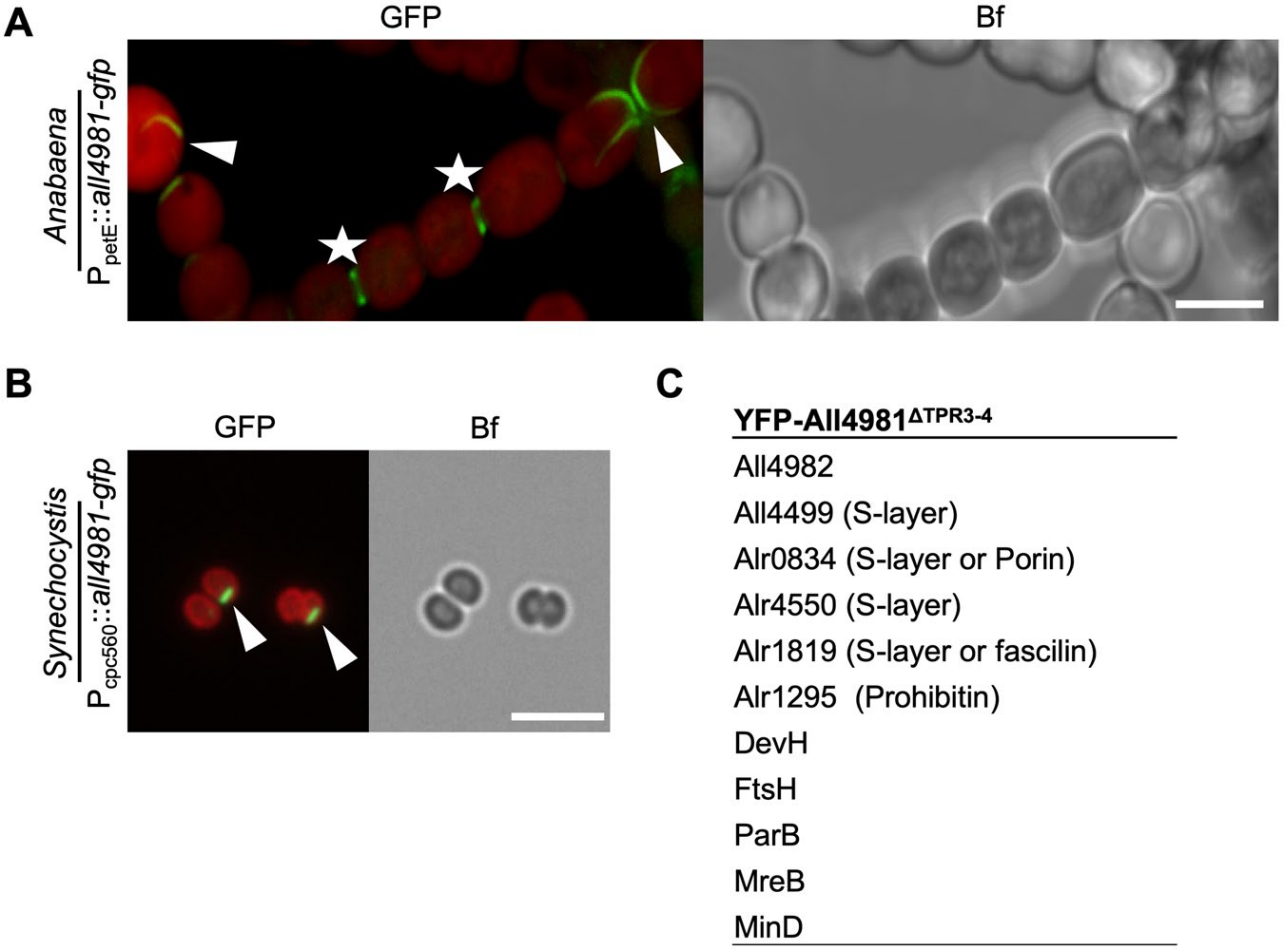
All4981 forms cell-traversing filament-like structures in cyanobacteria. (**A**,**B**) GFP fluorescence and merged GFP fluorescence and chlorophyll autofluorescence (red) and bright field micrographs of (**A**) *Anabaena* and (**B**) *Synechocystis* cells expressing All4981-GFP. *Anabaena* cells were grown in BG11_0_ and *Synechocystis* cells were grown in BG11. (**A**) Maximum intensity projections of a Z-stack. White triangles indicate selected filament-like strings traversing through the cells. White arrows point to spindle-like All4981-GFP structures. White stars mark septal formations between two neighboring cells. Scale bars: 5 µm. (**C**) Excerpt of interacting proteins of interest from mass spectrometry analysis of anti-GFP co-immunoprecipitations of *Anabaena* cells expressing YFP-All4981^ΔTPR3–4^ from P_petE_.

The expression of All4981-GFP in *Anabaena* revealed numerous filament-like structures that traversed the cell while in other cells All4981-GFP was associated with the cell septa (Fig. 5A). All4981-GFP filament-like structures also occasionally spread in a star-like pattern into the cytosol. Additionally, in freshly ruptured All4981-GFP-expressing cells, filament-like *ex vivo* structures assembled in the medium into an interconnected network (Supplementary Fig. 4F), resembling the *in vitro* polymerization pattern of All4981 (Fig. 2). We confirmed a host-independent *in vivo* polymerization capacity of All4981 by expressing All4981-GFP in *Synechocystis*, which lacks homologs to that protein (Fig. 5B). Intrigued by the septal localization, we tested for an interaction with SepJ, a septal junction protein in *Anabaena*^7^, and found weak, albeit significant physical interaction (Supplementary Fig. 3A). In addition, bacterial two-hybrid assays revealed that All4981 strongly interacted with the cell shape-determining protein MreB (Supplementary Fig. 3A). Notably, MreB has previously been shown to form similar filamentous structures in *Anabaena*. However, in contrast to genes in the *mreBCD* operon, whose overexpression induces abnormal cell morphologies^15^, no direct morphogenic influence was detected for All4981 in *Anabaena*. It is likely that All4981 is an essential protein in *Anabaena* as we were not able to generate an *all4981* deletion strain. Initially, we accidently also created a YFP-All4981 fusion construct with a deletion of 240 bp between nt 735 and nt 975 of the *all4981* CDS, resulting in a deletion of the third and fourth TPR (YFP-All4981^ΔTPR3–4^) leaving the remaining ORF intact. Remarkably, this fusion protein, like All4981-GFP, formed cell-traversing filament-like or spindle-like strings in *Anabaena* (Supplementary Fig. 4G). In contrast, full length YFP-All4981 localized to the septa between two neighboring cells but also revealed indistinct cytosolic localization (Supplementary Fig. 4G). Co-immunoprecipitation experiments following LC-MS/MS analytics from *Anabaena* WT expressing YFP-All4981^ΔTPR3–4^ revealed an association of YFP-All4981^ΔTPR3–4^ with ParB, MinD and MreB (Fig. 5C). Thus, All4981 might be involved in ParA/B/S-driven plasmid or chromosome segregation. The interaction with MreB agrees with the *in vivo* localization of YFP-All4981^ΔTPR3–4^ in *Anabaena* (Supplementary Fig. 4G) and the results from the bacterial two-hybrid assay (Supplementary Fig. 3A). Further interactions were found with a variety of putative S-layer and prohibitin-like proteins and with DevH, an essential protein for heterocyst glycolipid layer synthesis. Notably, we never observed All4981 expression in heterocysts, regardless of the fluorescence tag. All4981 also interacted with All4982, a protein encoded directly upstream of *all4981*, but not with All4983, which is encoded upstream of *all4982* (Supplementary Fig. 1C). This observation, together with the common transcript of *all4981* and *all4982* (Supplementary Fig. 1C) suggests a common function of both proteins. Thus, we attempted to localize All4982 with an eCFP tag in *Anabaena* but could not observe a coherent localization pattern. Overall, our results demonstrate that All4981 is connected to the MreB cytoskeleton, the septal junctions and the protective S-layer. Additionally, All4981 polymerizes *in vitro*, *in vivo* and *ex vivo*, is likely essential for *Anabaena* and is thus accordingly classified as a novel cyanobacterial TPR-repeat protein with the capacity to polymerize.

### *Synechococcus* CCRPs are involved in cytokinesis and colony integrity

**Figure 6:**
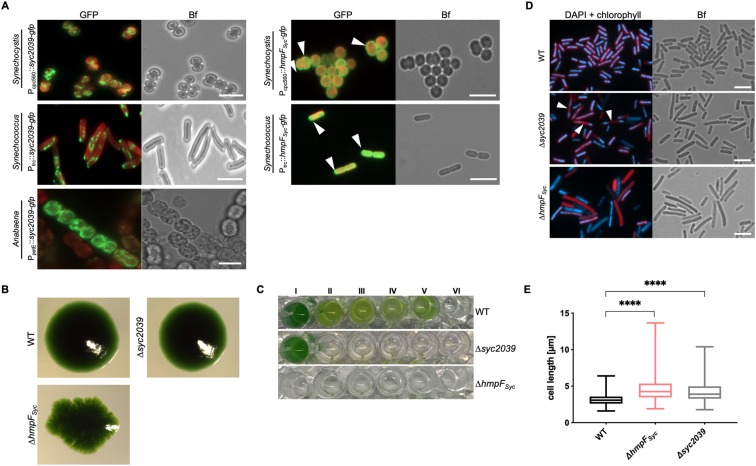
*Synechococcus* CCRPs affect cytokinesis and cellular integrity. (**A**) Merged GFP fluorescence and chlorophyll autofluorescence (red) and bright field micrographs of *Synechocystis*, *Synechococcus* and *Anabaena* cells expressing Syc2039-GFP or HmpF_Syc_-GFP from P_cpc560_, P_petE_ or P_trc_. *Synechocystis* cells were grown in BG11, *Anabaena* cells were grown in BG11_0_ supplemented with 0.25 µM CuSO_4_ for 1 day, and *Synechococcus* cells were grown on BG11 plates supplemented with 0.01 mM (Syc2039) or 1 mM (HmpF_Syc_) IPTG. Micrographs of *Synechococcus* and *Anabaena* cells expressing Syc2039-GFP are maximum intensity projections of a Z-stack. White triangles indicate HmpF_Syc_-GFP spots. Attempts to translationally fuse a YFP-tag to the N-terminus of Syc2039 were unsuccessful, possibly due to the transmembrane domain predicted to the Syc2039 N-terminus (Supplementary Table 1). (**B**) Colony formation of *Synechococcus* WT and mutant strains on BG11 plates. (**C**) Cell viability of *Synechococcus* WT and mutant strains grown in (I) BG11 or BG11 supplemented with (II) 5 mM glucose, (III) 200 mM glucose, (IV) 2 mM NH_4_Cl, (V) 200 mM maltose or (VI) 500 mM NaCl. (**D**) Merged DAPI fluorescence and chlorophyll autofluorescence (red) and bright field micrographs of *Synechococcus* WT and mutant strains grown on BG11 plates and stained with 10 μg ml^−1^ DAPI. White triangles indicate non-dividing cells revealing inhomogeneous DNA placement. (**E**) Cell length of *Synechococcus* WT (n = 648), non-segregated Δ*hmpF*_*Syc*_ (n = 417) and Δ*syc2039* (n = 711) mutant cells. Values indicated with * are significantly different from the WT. ****P < 0.0001 (one-way ANOVA, using Turkey’s multiple comparison test). Scale bars: 5 µm.

The results of the *in vivo* localization of a functional Syc2039-GFP fusion protein (Supplementary Fig. 6E,F) contrasted the ambiguous *in vitro* polymerization pattern (Fig. 2). Filament-like strings were readily observed in different cyanobacterial hosts, indicating that Syc2039 self-polymerization is independent of the host (Fig. 6A). However, Syc2039 formed different structures in each host. In *Anabaena*, the filament-like structures were long, curved and intertwined; in *Synechocystis* Syc2039-GFP appeared as spindle-like structures and in *Synechococcus* filament-like structures were long, sometimes helical and often aligned with or in close proximity to the cell envelope (Fig. 6A). A similar helical or cell periphery-aligned localization pattern was also observed in *E. coli* (Supplementary Fig. 1E). In *Synechocystis* and *Synechococcus* HmpF_Syc_-GFP localized as spots at the cell periphery, while in *E. coli* it seemingly coated the entire cell envelope (Fig. 6A, Supplementary Fig. 1E). HmpF_Syc_-GFP failed to be expressed in *Anabaena*, suggesting that (over-)expression of this protein has a negative impact on that organism. Using double homologous gene replacement, we generated a *Synechococcus* ∆*syc2039* mutant strain and a non-segregated *Synechococcus* ∆*hmpF*_*Syc*_ mutant strain (Supplementary Fig. 6A–C). The non-segregated nature of the ∆*hmpF*_*Syc*_ mutant suggests that this gene performs an essential cellular function and cannot be fully deleted. Colony integrity of the ∆*syc2039* mutant was unaltered while the ∆*hmpF*_*Syc*_ mutant was characterized by apparent changes in colony morphology (Fig. 6B), which were lost upon growth on non-selective plates (Supplementary Fig. 6D). Additionally, both mutants presented an impairment in liquid culture growth: the ∆*syc2039* mutant grew in standard BG11 medium but failed to grow upon addition of several osmotic stressors, whereas the ∆*hmpF*_*Syc*_ mutant failed to grow in liquid culture entirely (Fig. 6C). Spot assays confirmed a decreased viability of the ∆*hmpF*_*Syc*_ mutant and showed that it is highly sensitive to Proteinase K but unaffected by lysozyme (Supplementary Fig. 7A). These cell wall defects, together with the *in vitro* cell shape-like polymerization pattern suggest that HmpF_Syc_ might form a protective and protease-resistant proteinaceous layer below the cytoplasmic membrane. This possibility would also be in concert with the distorted colony morphology of the non-segregated ∆*hmpF*_*Syc*_ mutant strain. The Δ*syc2039* mutant was unaffected by cell wall and membrane destabilizers (Supplementary Fig. 7B). To investigate the role of these proteins in cell division, we stained intracellular DNA with DAPI and localization of FtsZ was detected by immunofluorescence in *Synechococcus* WT and both mutant strains. A proportion of ∆*syc2039* mutant cells exhibited a segregated DNA distribution either to both cell poles or to just one pole (Fig. 6D). Furthermore, some cells of both mutants lacked any discernible intracellular DNA or perceptible chlorophyll signal and were elongated compared to the WT (Fig. 6D,E). The WT phenotype of the ∆*syc2039* mutant could be rescued by insertion of P_trc_::*syc2039*-*gfp* or P_syc2039_::*syc2039* into the neutral NS1^52^ locus (Supplementary Fig. 6E,F). Although both mutant cells were elongated compared to WT cells (Fig. 6E), the intracellular localization of FtsZ was unaffected (Supplementary Fig. 7C). And despite the defect in cytokinesis, the Δ*syc2039* mutant strain revealed similar liquid culture growth properties as the WT (Supplementary Fig. 7D). Taken together, Syc2039 forms abundant filament-like networks *in vivo* and is involved in cytokinesis or cell cycle control. We could further show that *hmpF*_*Syc*_ is an essential gene important for cytokinesis, cellular integrity and colony formation, implicating structural functions.

## Discussion

Earlier studies suggested that there is likely a broad spectrum of coiled-coil-rich and rod-domain containing proteins with IF-like function in prokaryotes^29^. And indeed, reports on such proteins followed with the discovery of Scy (in *Streptomyces coelicolor*) and several CCRPs from *Helicobacter pylori*^30,31,34,35^. Here we further investigated the presence and function of CCRPs with filament-forming IF-like properties in prokaryotes, by predicting and evaluating CCPRs in cyanobacteria. Our *in vitro* polymerization assay allowed for a rapid detection of proteins with the potential to form filament-like structures *in vitro* using fluorescence microscopy. However, we note that for conclusive proof of *in vitro* filaments, higher resolution microscopy like electron microscopy would be necessary and will be addressed in future studies. The observed protein filament-like lengths were in the range of previously described *in vitro* filaments of FtsZ^53^ and of the human prion protein in its amyloid form^54^ that were obtained by a similar experimental procedure.

Our results show that Fm7001 assembles into polymers *in vitro* upon renaturation from urea as well as *in vivo*, and that this protein has an impact on cellular and trichome morphology, thereby fulfilling major IF criteria^40,55^. Consequently, we propose that Fm7001 constitutes a novel filament-forming CCRP specific to multicellular, cell-differentiating and branching cyanobacteria. The floating Fm7001 polymer sheet in high molar urea (i.e. 4.5 M urea) indicates an exceptionally high self-association capacity of Fm7001. In comparison, the eukaryotic IF protein Vimentin exists only as tetramers in 5 M urea^23^. *In vivo* localization experiments revealed an essential role of the Fm7001 C-terminus for polymerization, which is a common observation for known prokaryotic filament-forming proteins, including MreB^56^, Crescentin^25^ as well as eukaryotic IF proteins^23,57–60^. Additionally, the assigned structural similarities of Fm7001 with the acetyl-CoA-carboxylase may provide further support for the theory that filament-forming proteins originated from metabolic enzymes that obtained polymerization features^61^. Notwithstanding, the metabolic activity of Fm7001 was not evaluated in our study hence its presumed enzymatic activity remains to be tested. Additionally, so far, no sufficient genome modification systems exist for *Fischerella*^62,63^, as such a precise analysis of the function of Fm7001 is currently not possible.

Several prokaryotic tubulin-like and actin-like cytoskeletal proteins, such as ParM and TubZ, are known to be encoded on plasmids or on bacteriophages^9,64^. In *Synechocystis*, *slr7083* is encoded on the large toxin-antitoxin defense plasmid (pSYSA)^65^, thus it adds another protein to the list of those CCRPs with the property to assemble into filament-like structures carried by an autonomously replicating genetic element. Preliminarily, we suspected that Slr7083 has a role in plasmid-segregation similar to ParM. However, Slr7083 showed no indications of dynamic properties, which would be indispensable for a plasmid segregation mechanism. As Slr7083 was ectopically overexpressed, this observation has to be taken with a grain of salt as overexpression could interrupt the protein dynamics. Furthermore, unlike ParM^66^, Slr7083 did not localize in a spindle-like pattern *in vivo* and was only expressed at later growth phases, which is contradictory to a possible involvement in the cell cycle. In contrast, the polymers formed by Slr7083 *in vitro* and *in vivo* rather suggest that it could form a proteinaceous layer below the cytoplasmic membrane. Notably, Slr7083 *in vitro* structures resemble the nuclear lamina formed by nuclear lamins and FilP lace-like *in vitro* filaments^29,67,68^. It is thus conceivable that Slr7083 has a role in cellular stiffness as well as rigidity and mediates mechanical cell stabilization. However, restriction of transcription to only a comparably short period of the culture growth phase challenges the idea of a cell-stabilizing function for Slr7083. In contrast, cell motility in *Synechocystis* seems to be partially regulated by Slr7083, reminiscent of the role of the actin cytoskeleton in eukaryotes.

The role of Slr7083 in cell motility is possibly mediated by means of its interaction with HmpF_Syn_, which has been previously shown to be essential for twitching motility in *Synechocystis*^50^. So far it is unknown how photoreceptors transduce the perceived light stimuli to the motility apparatus in *Synechocystis* ultimately resulting in phototactic movements^69^. It is tenable to hypothesize that HmpF_Syn_ might constitute the missing link between the two systems, possibly in combination with Slr7083. This hypothesis is supported by the physical interaction of HmpF_Syn_ with PilB and the *in vivo* localization of HmpF_Syn_ that is similar to that observed for PilB^49^. Direct interaction of HmpF_Syn_ and PilB provides further support for the model from Cho *et al*.^51^ according to which, a direct interaction of HmpF with PilB could activate the pilus extension by the type IV system. In *N. punctiforme*, HmpF was found to dynamically localize to the leading or lagging pole of hormogonia depending on the light intensity and in a dependent manner with the type IV apparatus and the Ptx system^51^. In *Synechocystis*, localization of PilB was shown to correlate with the direction of movement^49^ and to be dependent on the direction of incoming light in a complex with other pili protein^70^. Given its similar localization with PilB, we hypothesize that like HmpF from *N. punctiforme*, HmpF_Syn_ may be characterized by dynamic light-dependent properties in a complex with the type IV pilus system. A similar motility complex was observed in *Pseudomonas aeruginosa*, where FimL (a proposed scaffolding protein with a weakly predicted coiled-coil) was shown to connect the chemosensory receptor system to the type IV pili apparatus, regulating the chemotactic and virulence pathways^71^. In eukaryotes, cellular motility is strongly dependent on cytoskeletal proteins^72^, thus it is possible that filament-forming proteins are also key factors for cell locomotion in prokaryotes. Although IFs do not directly participate in cell motility in eukaryotes^73^, an adaptation of filament-forming CCRPs in prokaryotes for this task is conceivable. Bactofilins constitute a separate class of prokaryotic-specific polymerizing proteins and were proposed to be involved in coordinated motility in *C. crescentus*^43^. Additionally, the filament-forming CCRP AglZ from *Myxococcus xanthus* was previously shown to govern gliding motility together with a multi-protein complex that also involves the MreB cytoskeleton^74,75^. Notably, the interaction of HmpF_Syn_ with FtsZ and MinD, which are essential cellular factors involved in cell division, cytokinesis and Z-ring placement^76^, might indicate that HmpF_Syn_ function is not restricted to motility. Although thylakoid membranes pose no physical barrier for proper Min system oscillation in *Synechococcus*, suggesting no direct interaction, MinD is part of the thylakoid fractions in *Synechocystis*^77^. In concert with its localization to the cell envelope in *Synechocystis*, it would be intriguing to investigate a potential link between HmpF_Syn_ and thylakoid development and cytokinesis during cell division. Despite their different cellular functions and *in vitro* polymerization properties, the homologous proteins HmpF_Syn_, HmpF_Syc_ and HmpF_Ana_ retained the ability to cross-interact (Supplementary Fig. 3A). Further studies may focus on identifying the protein domains that mediate this interaction, likely residing within the highly conserved amino acid sequence region in this homologous protein family (Supplementary Fig. 3B). These regions are likely important for an interaction with species-specific proteins that lead to their species-specific cellular function.

TPR proteins are known to mediate protein-protein interactions and can assemble into multimers, but their ability to polymerize into filaments has not been described so far^78^. Nonetheless, All4981 polymerized *in vitro* and formed filament-like structures *in vivo* in all tested hosts. Additionally, it forms extracellular filament-like structures and is presumably an essential protein in *Anabaena*. These observations suggest that All4981 is a *bona fide* prokaryotic TPR protein with the property of filament-like assembly. The association of All4981 with MreB, FtsZ-regulators, the S-layer and SepJ indicates that it might function as a bridge that connects the shape-determinants outside of the cell wall and inside of the cytoplasmic membrane to the sites of cell-cell connections (i.e. septal junctions).

Considering the presence of an N-terminal transmembrane domain and the lack of clear *in vitro* filaments, it is unlikely that Syc2039 constitutes a genuine filament-forming protein. Nonetheless, the highly abundant filament-like network structures formed in all tested bacterial hosts suggests that Syc2039 is associated with cytoskeletal structures. Specifically, the elongated phenotype and the disturbed cytokinesis in the *Synechococcus* Δ*syc2039* mutant and the non-segregated Δ*hmpF*_*Syc*_ mutant suggest an association with the FtsZ-driven divisome. Direct interaction with FtsZ or MreB could not be shown, as such, future studies will likely attempt to unravel the presumed connection of the *Synechococcus* CCRPs to those two major cytoskeletal systems. Notably, besides its cytokinetic defect, the Δ*syc2039* mutant showed growth characteristics like the WT, suggesting that feedback mechanisms between cytokinesis and cell division are disturbed in the Δ*syc2039* mutant.

**Figure 7:**
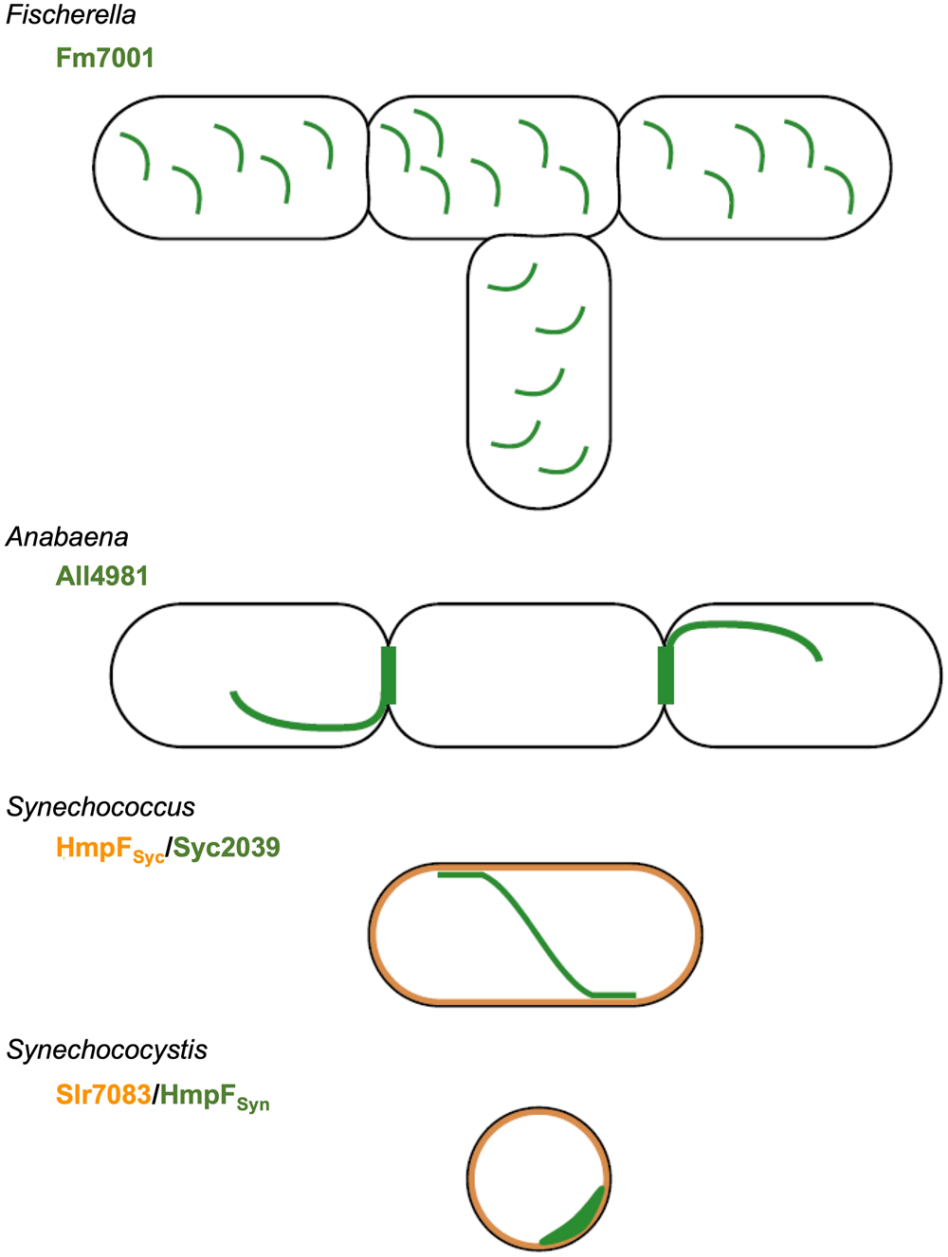
Cyanobacterial CCRP systems. Schematic models for the *in vivo* localization of cyanobacterial CCRPs in their respective hosts. Fm7001 forms filament-like strings in *Fischerella*. In *Anabaena*, All4981 assembles into pole-arising filament-like structures that traverse through the cell or forms septal-localized bridge-like formations. Syc2039, either independently of other *Synechococcus* proteins, or in direct cooperation with other putative filamentous proteins, forms long and sometimes helical strings that are often aligned with or in close proximity to the cell periphery. In *Synechococcus*, HmpF_Syc_ likely forms a protective proteinaceous layer below the cytoplasmic membrane. In *Synechocystis*, HmpF_Syn_ forms crescent-like structures while Slr7083 seemingly underlies the cytoplasmic membrane. Both localization types were also observed for PilB, suggesting a cooperative function.

Our results reveal two novel filament-forming CCRPs - Fm7001 and All4981 - from different cyanobacterial subsections and morphotypes (Fig. 7). Our study thus extends the spectrum of known filament-forming CCRPs in prokaryotes and expands the set of functional properties associated with IF-like proteins in prokaryotes. As previously suggested^29^, we demonstrate that the sole observation of coiled-coil-rich regions within a protein sequence cannot be regarded as a sole predictor of protein polymerization, hence identification of novel filament-forming proteins requires additional *in vitro* and *in vivo* assays. The cyanobacterial CCRPs we report here, like other bacterial CCRPs^25,29,30,34,35,79^ and eukaryotic IFs^80^ are essential cellular components (All4981), are important for cell shape determination (Fm7001, HmpF_Syc_ and Syc2039), mediate cellular motility (Slr7083 and HmpF_Syn_), DNA segregation (HmpF_Syc_ and Syc2039) and colony integrity (HmpF_Syc_). Our study thus strengthens the perception that like eukaryotes, prokaryotes require organized internal complexes and even microcompartments to maintain cell shape, size and proper cell function and highlights the usefulness of polymerized proteinaceous structures for cellular processes. Remarkably, some of the identified CCRPs were highly conserved among all cyanobacterial morphotypes, suggesting that their function is conserved. Future studies are required in order to evaluate the functional conservation of homologous proteins in different cyanobacterial species and morphotypes. On the other hand, Syc2039 and Slr7083 are highly strain specific, possibly performing a function that is adapted to the very needs of their hosts. Similarly to the eukaryotic cytolinker proteins^81,82^, cyanobacterial CCRPs were often associated with other cytoskeletal systems (MreB, FtsZ and other filament-forming CCRPs) and sites of cell-cell connections (i.e. SepJ), which demonstrates the necessity for those structures to be in a constant interplay even in comparably small cells. The discovery of filament-forming CCRPs with different levels of conservation in various cyanobacterial morphotypes thus opens up new avenues of research on their contribution to cyanobacterial morphological diversity.

## Material and Methods

### Data and CCRP prediction

The cyanobacterial protein families were constructed from completely sequenced genomes available in RefSeq database^83^ (V. May 2016; Supplementary File 3). For the construction of protein families, at the first stage, all protein sequences annotated in the genomes were blasted all-against-all using stand-alone BLAST^84^ (V. 2.2.26). Protein sequence pairs that were found as reciprocal best BLAST^85^ hits (rBBHs) with a threshold of E-value ≤ 1 × 10^−5^ were further compared by global alignment using needle^86^ (EMBOSS package, V. 6.6.0.0). Sequence pairs having ≥30% identical amino acids were clustered into protein families using the Markov clustering algorithm (MCL)^87^ (V. 12–135) with the default parameters. For the CCRPs prediction, 1,535 protein sequences containing non-standard amino acids were discarded. Coiled-coil regions in protein sequences were predicted using PEPCOIL^86^ (EMBOSS package, V. 6.6.0.0). The algorithm was executed with a window size of 21 and the threshold for amino acids in coiled-coil conformation was set to ≥80 amino acid residues similarly as described previously^29^. Statistical tests were performed with MatLab©. For the comparison of CCRPs proportion, the compared groups included: (1) SynProCya group, (2) unicellular cyanobacteria, (3) unicellular cyanobacteria that divide in more than one plane, and (4) multicellular cyanobacteria. Identification of conserved amino acid domains within cyanobacterial CCRP homologs (HmpF_Syn_ (Slr1301) and HmpF_Syc_ (Synpcc7942_1139)) was done using MULTALIGN^88^.

Protein candidates were further manually examined with online available bioinformatic tools (NCBI Conserved Domain (CD) Search^89^, TMHMM Server^90^ (V. 2.0), PSIPRED^91^, PSORTb^92^ (V. 3.0), I-TASSER^93^. CCRPs exhibiting similar predictions to known IF and IF-like proteins like CreS, FilP, Vimentin, Desmin or Keratin were selected, and proteins predicted to be involved in other cellular processes were excluded.

### Bacterial strains and growth conditions

*Fischerella*, *Anabaena* and *Synechocystis* were obtained from the Pasteur Culture Collection (PCC) of cyanobacteria (France). *Synechococcus* was a gift from Martin Hagemann (University Rostock). Glucose-tolerant motile *Synechocystis* PCC-M substrain was a gift from Annegret Wilde (University Freiburg). Cells were grown photoautotropically in BG11 or without combined nitrogen (BG11_0_) at a 16 h/8 h light/dark regime (*Fischerella*) or at constant light (*Anabaena*, *Synechococcus* and *Synechocystis*) with a light intensity of 20 µmol m^−2^ s^−1^. When appropriate, 50 µg ml^−1^ kanamycin (Km), 2.5 µg ml^−1^ spectinomycin (Sp), 2.5 µg ml^−1^ streptomycin (Sm) or 30 µg ml^−1^ neomycin (Nm) was added. Non-segregated Δ*hmpF*_*Syc*_ cells were always grown in the presence of Km. *E. coli* strains DH5α, DH5αMCR, XL1-blue and HB101 were used for cloning and conjugation by triparental mating. BTH101 was used for BACTH assays and BL21 (DE3) was used for expression of His- and GFP-tagged proteins in *E. coli*. All *E. coli* strains (Supplementary Table 2) were grown in LB medium containing the appropriate antibiotics at standard concentrations.

### Plasmid and strain construction

All plasmids employed in this study were either generated by using standard restriction enzyme-base cloning procedures or using Gibson assembly^94^. A detailed description of the cloning strategies for the respective plasmids is available upon request from the authors. All primers, plasmids and strains employed or generated in this study are listed in Supplementary Tables 2–5. GFP, YFP and eCFP protein tags were used as reporter proteins and His_6_ tag was used for protein affinity purification. For gene replacement mutants, homologous flanks for double homologous recombination comprised 1000 bp upstream and downstream of the gene of interest. Mutant strains harboring gene replacements with antibiotic resistance cassettes (*nptII*^95^ or *CS.3*^96^) were verified by colony PCR testing for absence of gene of interest using primers #129/#130 for Δ*slr7083*, primers #168/#169 for Δ*hmpF*_*Syn*_, primers #146/#147 for Δ*syc2039* or primers #161/#162 for Δ*hmpF*_*Syc*_. We also attempted to generate gene replacement mutants for *all4981* and *fm7001* but remained unsuccessful.

### Transformation of cyanobacteria

Transformation of *Synechococcus* was achieved by natural transformation as described previously^97^ and transformation of *Synechocystis* was accomplished by natural transformation or by conjugation as described previously^98,99^. *Anabaena* and *Fischerella* were transformed by conjugation as previously described^62,99^. Ex-conjugant colonies from *Synechococcus* and *Synechocystis* carrying gene replacements were re-streaked three to four times and absence of genes of interest was verified by colony PCR. Transformation of sonicated (fragmented) and NaCl-treated *Fischerella* cells followed by the conjugational method^99^ was also feasible for *Fischerella*, albeit with a lower transformation frequency.

### Phenotypic characterization of the mutant strains

Defects in cell viability were evaluated by spot assays adapted from Dörrich *et al*. (2014)^100^. Wild type and mutant strains from liquid cultures or BG11 plates were adjusted to an OD_750_ of about 0.4 in liquid BG11 liquid. Next, 5 µl of cells were spotted in triplicates onto BG11 plates or BG11 plates supplemented with Proteinase K or lysozyme at indicated concentrations in 10-fold serial dilutions and incubated under standard growth conditions until no further colonies arose in the highest dilution.

Growth defects were assessed with growth curves. For this, cells were grown in liquid BG11 medium, washed three times by centrifugation (6500 × *g*, RT, 3 min) in BG11, adjusted to an OD_750_ of 0.1 and then grown in triplicates or quadruples at standard growth conditions in 15 ml culture volumes. OD_750_ values were recorded every 24 h.

Cell length of *Synechococcus* WT, mutant strains and mutant complementation strains was measured using the line tool from the imaging software Fiji and its plugin microbeJ.

Cell wall integrity defects were evaluated by testing the influence of osmotic factors on cell growth. *Synechococcus* WT and mutant strains were grown on BG11 agar plates, transferred to BG11 liquid medium and grown under standard growth conditions with or without 5 mM glucose, 200 mM glucose, 2 mM NH_4_Cl, 200 mM maltose or 500 mM NaCl.

To evaluate the motility of *Synechocystis* and PCC-M WT and mutant strains, three single colonies of the respective strain were streaked on a line on a BG11 growth plate. Growth plates were then placed into the standard culture incubator for 10 d with illumination limited from one direction.

### Protein purification and *in vitro* filamentation assays

For protein purification, *E. coli* BL21 (DE3) cells carrying His_6_-tagged protein candidates were grown in overnight cultures at 37 °C and 250 rpm. The next day, overnight cultures were diluted 1:40 in the same medium and grown at 37 °C until they reached an OD_600_ of 0.5–0.6. Protein expression was induced with 0.5 mM IPTG for 3–4 h at 37 °C and 250 rpm. Afterwards, cell suspensions of 50 ml aliquots were harvested by centrifugation, washed once in PBS and stored at −80 °C until further use. For *in vitro* filamentation assays, cell pellets were resuspended in urea lysis buffer (ULB: 50 mM NaH_2_PO_4_, 300 mM NaCl, 25 mM imidazole, 6 M urea; pH 8.0) and lysed in a Precellys® 24 homogenizer (3 × 6500 rpm for 30 s) using the 2 ml microorganism lysis kit (VK01; Bertin) or self-packed Precellys tubes with 0.1 mm glass beads. The resulting cell debris was pelleted by centrifugation at 21,000 × *g* (30 min, 4 °C) and the supernatant was incubated with 1 ml HisPur™ Ni-NTA resin (Thermo Fischer Scientific) for 1 h at 4 °C in an overhead rotator. The resin was washed 5 times with 4x resin-bed volumes ULB and eluted in urea elution buffer (UEB: ULB supplemented with 225 mM imidazole). Total protein concentration was measured using the Qubit® 3.0 Fluorometer (Thermo Fischer Scientific) and generally adjusted to 0.5–1.0 mg ml^−1^ before dialysis. Purified proteins were dialyzed overnight against polymerization buffer (PLB: 50 mM PIPES, 100 mM KCl, pH 7.0; HLB: 25 mM HEPES, 150 mM NaCl, pH 7.4) at 18 °C and 180 rpm with three bath changes using a Slide-A-Lyzer™ MINI Dialysis Device (10 K MWCO, 0.5 ml or 2 ml; Thermo Fischer Scientific). Purified proteins were stained with 0.005 mg NHS-Fluorescein (Thermo Fischer Scientific) per 1 ml protein dialysate and *in vitro* filamentation was analyzed by epifluorescence microscopy.

For Fm7001-His_6_, proteins were slowly dialyzed against 2 mM Tris-HCl, 4.5 M urea, pH 7.5 (18 °C, 200 rpm) decreasing 0.5 M urea every 2 h (from 6 M to 4.5 M urea). The resulting floating filament-like web was then analyzed by bright field microscopy.

Syc2039-His_6_ failed to be expressed in *E. coli* BL21 (DE3). To bypass this, Syc2039-GFP-His, under the control of an IPTG-inducible P_trc_, was inserted into a neutral locus of *Synechococcus*. Cells were grown to an OD_750_ of 0.8 and protein expression was induced with 0.05 mM IPTG for 3 d. Induced cells were harvested and washed with PBS by centrifugation (4800 × *g*, 4 °C, 10 min) and stored at −80 °C. Protein purification, dialysis and labeling was then performed as described above with the exception that BG11 growth medium was used as dialysate.

### Co-immunoprecipitation

For co-immunoprecipitations of fluorescently tagged CCRP candidates, cyanobacterial strains expressing YFP-All4981 or HmpF_Syn_-YFP were grown in BG11 or BG11_0_ liquid medium. About 20–30 ml of the respective culture was pelleted by centrifugation (4800 × *g*, 10 min, RT), cells were washed twice by centrifugation (4800 × *g*, 10 min, RT) with 40 ml PBS and then resuspended in 1 ml lysis buffer (PBS-N: PBS supplemented with 1% NP-40) supplemented with protease inhibitor cocktail (PIC; cOmplete^™^, EDTA-free Protease Inhibitor Cocktail, Sigma-Aldrich). Cells were lysed using the VK05 lysis kit (Bertin) in a Precellys® 24 homogenizer (3 strokes for 30 seconds at 6500 rpm) and cell debris was pelleted by centrifugation (30 min, 21,100 × *g*, 4 °C). 50 µl μMACS anti-GFP MicroBeads (Miltenyi Biotec) was added to the resulting cell-free supernatant and incubated for 1 h at 4 °C with mild rotation. Afterwards, the sample was loaded onto µColumns (Miltenyl Biotec), washed two times with 1 ml lysis buffer and eluted in 50 µl elution Buffer (50 mM Tris HCl pH 6.8, 50 mM DTT, 1% SDS, 1 mM EDTA, 0.005% bromphenol blue, 10% glycerol; Miltenyl Biotec). Until further use, samples were stored at −80 °C. Proteins were identified by mass spectrometry. A detailed protocol of the mass spectrometry analysis is available upon request from the authors.

### Immunofluorescence

The localization of FtsZ in *Synechococcus* WT and mutant strains was evaluated by immunofluorescence using a modified protocol from Heinz *et al*.^101^. In contrast, cells were lysed in 50 mM Tris-HCl pH 7.4, 10 mM EDTA and 0.2 mg ml^−1^ lysozyme for 30 min at 37 °C and samples were blocked in 1x Roti®-ImmunoBlock (Carl Roth) in PBS supplemented with 0.05% Tween 20. Samples were incubated with rabbit anti-FtsZ primary antibody (Agrisera; raised against *Anabaena* FtsZ; 1:250 diluted) in blocking buffer followed by incubation with 7.5 µg ml^−1^ Alexa Fluor 488-conjugated goat anti-rabbit IgG (H + L) secondary antibody (Thermo Fischer Scientific) in blocking buffer. Before microscopy, cells were stained with 10 µg ml^−1^ DAPI (final concentration) in PBS.

### Brightfield and fluorescence microscopy analysis

Bacterial strains grown in liquid culture were either directly applied to a microscope slide or previously immobilized on a 2% low-melting agarose in PBS agarose pad and air dried before microscopic analysis. Epifluorescence microscopy was performed using an Axio Imager.M2 light microscope (Carl Zeiss) equipped with Plan-Apochromat 63×/1.40 Oil M27 objective and the AxioCam MR R3 imaging device (Carl Zeiss). GFP, Alexa Fluor 488, eCFP and YFP fluorescence was visualized using filter set 38 (Carl Zeiss; excitation: 470/40 nm band pass (BP) filter; emission: 525/50 nm BP). Chlorophyll auto-fluorescence was recorded using filter set 15 (Carl Zeiss; excitation: 546/12 nm BP; emission: 590 nm long pass). When applicable, cells were previously incubated in the dark at RT for about 5 min with 10 µg ml^−1^ DAPI in PBS to stain intracellular DNA. For visualization of DAPI fluorescence filter set 49 (Carl Zeiss; excitation: G 365 nm; emission: 455/50 nm) was employed. *E. coli* BL21 (DE3) cells expressing C-terminally GFP-tagged protein candidates were grown over night in LB and then diluted 1:40 in the same medium the following day. Cells were grown for 2 h at 37 °C, briefly acclimated to 20 °C for 10 min and induced with 0.05 mM IPTG at 20 °C. Protein localization of GFP/YFP-tagged proteins was then observed after indicated time points of cells immobilized on an agarose pad.

### Statistical analysis

Beta-galactosidase values were measured in triplicates from three independent colonies and significant differences compared to WT were determined by a one-way ANOVA using Dunnett’s multiple comparison test. For statistical evaluation of *Synechococcus* WT and mutant cell length, a one-way ANOVA using Turkey’s multiple comparison test was used. Significance levels are the same as for the beta-galactosidase assay. Statistical tests were performed with the GraphPad Prims 8.0.0 software. Significance levels are indicated by stars (*) and correspond to: *P < 0.05, **P < 0.01, ***P < 0.001, ****P < 0.0001.

### RNA isolation and RT-PCR

Total RNA was isolated from 10 ml culture using either the Direct-zol™ RNA MiniPrep Kit (Zymo Research; *Synechocystis*, *Synechococcus* and *Anabaena*) according to the manufacturer’s instructions or the Plant RNA Reagent (Thermo Fischer Scientific; *Anabaena*, *Fischerella* and *Synechocystis*). For RNA isolation using the Plant RNA Reagent, a modified protocol was employed. To this end, cells were pelleted by centrifugation (4800 × *g*, 10 min, 4 °C) and the supernatant was discarded. The pellet was resuspended in 0.5 ml of Plant RNA Reagent und lysed in a Precellys® 24 homogenizer (Bertin) with 3 strokes at 6500 rpm for 30 s in 2 ml soil grinding (SK38) or tough microorganism (VK05) lysis tubes (Bertin). RNA was then isolated according to the manufacturer’s instructions. Isolated RNA was treated with DNA-free™ Kit (2 units rDNAs/reaction; Thermo Fischer Scientific) and 1 µg (*Fischerella*, *Synechocystis* and *Synechococcus*) or 200 ng (*Anabaena*) RNA was reverse transcribed using the Maxima™ H Minus cDNA Synthesis Master Mix (with dsDNase; Thermo Fischer Scientific, for *Fischerella*, *Synechocystis* and *Synechococcus*) or the qScript™ cDNA Synthesis Kit (Quanta Biosciences, for *Anabaena*). RT-PCR of cDNA samples for *fm7001*, *ftsZ*, *slr7083*, *rnpB*, *hmpF*_*Syn*_, *syc2039*, *hmpF*_*Syc*_, *all4981*, *all4981* + *all4982* and *all4981* + *all4983* was done using primer pairs #1/#2, #3/#4, #5/#6, #7/#8, #9/#10, #11/#12, #13/#14, #15/#16, #17/#15 and #18/#15, respectively.

### Bacterial two hybrid assays

In this study, the BACTH system (Euromedex) was employed. Gene candidates were cloned into the expression vectors pKNT25, pKT25, pUT18 and pUT18C by GIBSON assembly, thereby generating C and N-terminal translational fusions to the T25 or T18 subunit. Chemically competent *E. coli* BTH101 (Δ*cya*) cells were co-transformed with 5 ng of the indicated plasmids, plated onto LB plates supplemented with 200 µg ml^−1^X-gal, 0.5 mM IPTG, Amp, Km and grown at 30 °C for 24–36 h. Interactions were quantified by beta-galactosidase assays from three colonies for each combination according to the protocol described by Euromedex or in a 96 well format^102^. For this aim, cultures were either grown over night at 30 °C or for 2 d at 20 °C in LB Amp, Km, 0.5 mM IPTG and interaction strength of the investigated proteins was by quantified by beta-galactosidase-mediated hydrolyzation of ONPG (ortho-Nitrophenyl-β-galactoside), which is then recorded in Miller units^103^.

## Supplementary information

Supplementary Materials.

Supplementary File 1.

Supplementary File 2.

Supplementary File 3.

## Data Availability

The datasets generated during and/or analyzed during the current study are available from the corresponding authors on reasonable request.
