# Supplementary material for: Identification and characterization of novel filament-forming proteins in cyanobacteria
Source: Sci Rep. 2020 Feb 5;10:1894. doi: 10.1038/s41598-020-58726-9 (PMC7002697; doi:10.1038/s41598-020-58726-9)
Supplement: Supplementary file 1 — Supplementary Materials. [file 41598_2020_58726_MOESM1_ESM.pdf]

Supplementary Materials for

Identification and characterization of novel filament-forming proteins in cyanobacteria

Benjamin L. Springstein*, Christian Woehle, Julia Weissenbach, Andreas O. Helbig, Tal Dagan, Karina Stucken*

*Corresponding authors: BLS: benjamin_springstein@hms.harvard.edu
KS: kstucken@userena.cl

Supplementary Information

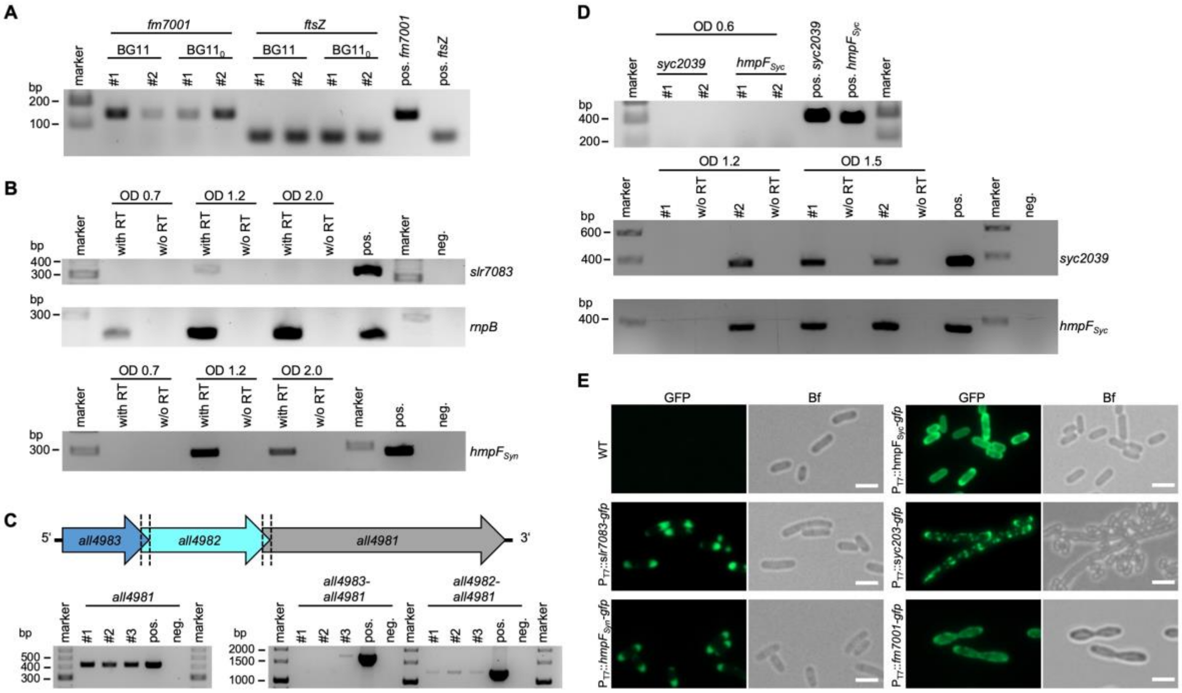

Supplementary Fig. 1: Expression of candidate CCRPs and heterologous expression in *E. coli*

(**A**) RT-PCR of reverse transcribed whole RNA from young *Fischerella* WT cultures grown in BG11 or BG11$_0$ from two independent biological replicates. Gene transcripts were verified using internal *fm7001* gene primers (#1/#2) or internal *ftsZ* gene primers (#3/#4) as a control. (**B**) RT-PCR of reverse transcribed whole RNA from *Synechocystis* WT (OD_{750} 0.7, 1.2 or 2.0) grown in BG11 using internal *slr7083* gene primers (#5/#6) or internal *hmpF*$_{Syn}$ gene primers (#9/#10). Internal *rnpB* gene primers (#7/#8) were included as a control. (**C,** top) Schematic representation of the genomic context of *all4981*. The 3' end of *all4983* overlaps with 4 bp with the 5' region of *all4982*, which has the same overlap with the 5' end of *all4981*. Both overlaps are comprised of the same four nucleotides (ATGA). (**C**, bottom) RT-PCR of reverse transcribed whole RNA from *Anabaena* WT cultures grown in BG11 (OD_{750} 1.8) from three independent biological replicates. *all4981* gene transcript was internal gene primers (#15/#16). For operon structure of *all4983-all4981* or *all4982-all4981*, primer pairs #17/#16 or #18/#16 were used, respectively. Only one replicate showed a common transcript for an *all4983-all4981* operon, which is likely is the result of the long fragment (about 1800 bp). The employed cDNA synthesis kit is optimized for fragments up to 1000 bp, thus making longer reverse transcriptions unlikely. (**D**) RT-PCR of reverse transcribed whole RNA from *Synechococcus* WT (OD_{750} 0.6, 1.2 or 1.5) grown in BG11 from two independent biological replicates. Gene transcripts were verified using internal *syc2039* gene primers (#11/#12) and internal *hmpF*$_{Syc}$ gene primers (#13/#14). (**B**,**D**) RNA was either reverse transcribed in the reaction buffer containing reverse transcriptase (with RT) or without reverse transcriptase (w/o RT) as a control for residual genomic DNA contamination. (**A**-**D**) Genomic DNA of the respective species was included as positive control for the different reactions. Full gel pictures as shown in Extended Data Supplementary Fig. 1. (**E**) GFP fluorescence and bright field micrographs of *E. coli* BL21 (DE3) cells expressing Slr7083-GFP, HmpF$_{Syn}$-GFP, HmpF$_{Syc}$-GFP, Syc2039-GFP or Fm7001-GFP. Cells were grown at 20 °C or (Fm7001-GFP) 16 °C and protein expression was induced with 0.05 mM IPTG for 24 h. Scale bars: 2.5 µm.

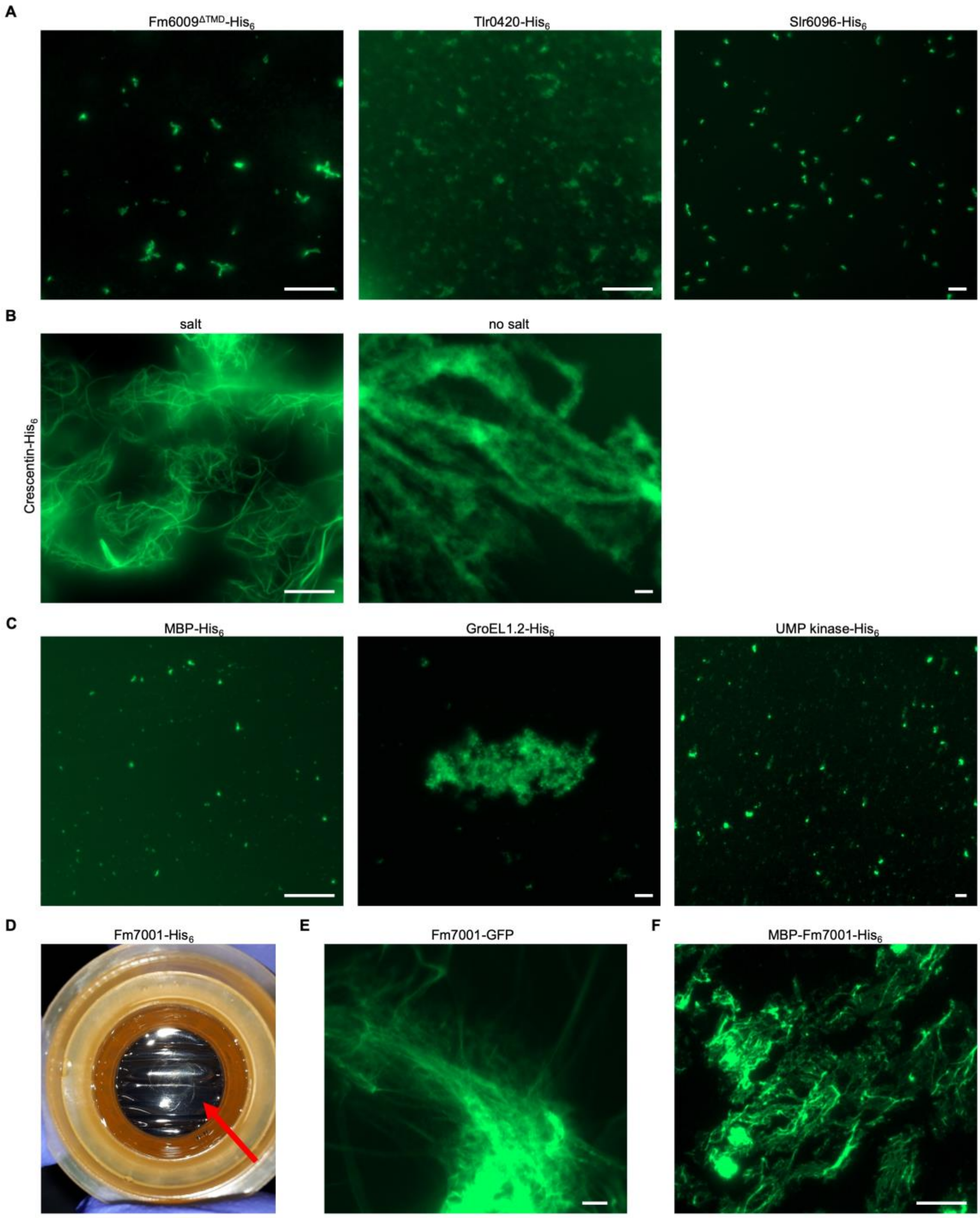

Supplementary Fig. 2: *In vitro* polymerization is dependent on monovalent ions

(A-C) NHS-fluorescein fluorescence micrographs of *in vitro* structures formed by purified and renatured Fm6009$_{\Delta TMD}$-His$_6$ (lacking the transmembrane domain, i.e. the first 91 aa), Tlr0420-His$_6$ or Slr6096-His$_6$ (1 mg ml^{-1} each), Crescentin-His$_6$ (0.7 mg ml^{-1}), MBP-His$_6$ (1 mg ml^{-1}), GroEL1.2 (0.7 mg ml^{-1}) or UMP kinase (0.5 mg ml^{-1}) , in HLB or Crescentin-His$_6$ (0.7 mg ml^{-1}) renatured in 25 mM Hepes, pH 7.4. Note: Crescentin-His$_6$ *in vitro* polymerization into smooth filaments is strictly dependent of the presence of salt in the renaturation buffer as Crescentin-His$_6$ without salt assembles into filamentous aggregates only. (**A**) Proteins were dialyzed in a stepwise urea-decreasing manner and stained with an excess of NHS-Fluorescein. (**D**) Bright field micrograph of a sheet-like flat object floating on top of the dialysate (red arrow) formed upon dialysis of Fm7001-His$_6$ (0.7 mg ml^{-1}) into 2 mM Tris-HCl, 4.5 M urea, pH 7.5. (**E,F**) Epifluorescence micrographs of filament-like structures formed by (**E**) denatured cell-free extracts of *E. coli* BL21 (DE3) expressing Fm7001-GFP (0.7 mg ml^{-1} whole protein) dialyzed into 2 mM Tris-HCl, 3 M urea, pH 7.5 or by (**F**) natively purified MBP-Fm7001-His$_6$ (0.8 mg ml^{-1}) stained with NHS-fluorescein in HLB. Scale bars: 10 µm.

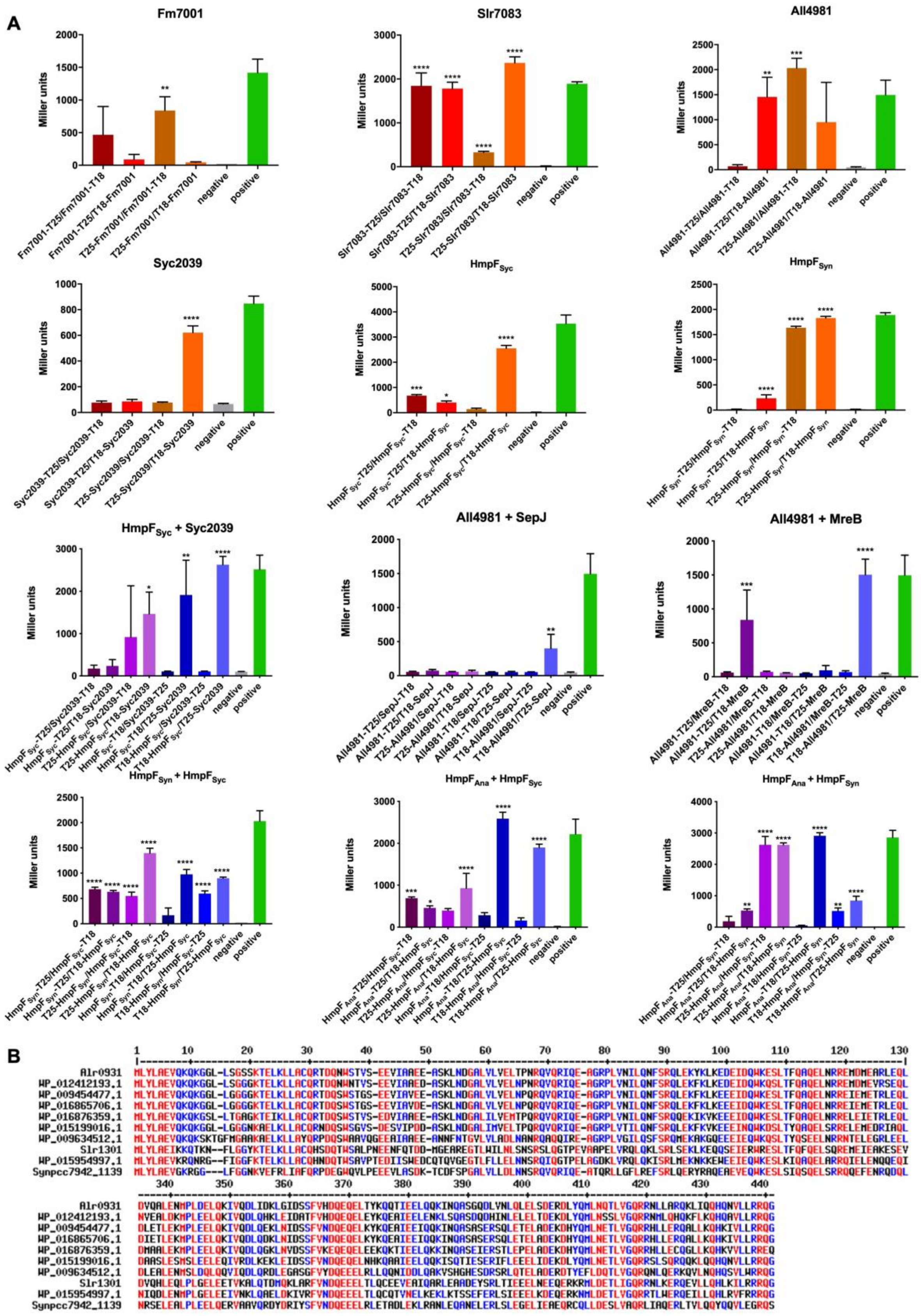

Supplementary Fig. 3: BACTH analysis of cyanobacterial CCRPs

(**A**) Beta-galactosidase assays (BACTH) of *E. coli* BTH101 cells co-expressing indicated T25 and T18 translational fusions of all possible pair-wise combinations from three independent colonies grown for 1 d at 30 °C or 2 d at 20 °C. Quantity values are given in Miller Units per milligram LacZ of the mean results from three independent colonies. Negative: N-terminal T25 fusion construct of the respective protein co-transformed with empty pUT18C.

Positive: Zip/Zip control. Error bars indicate standard deviations (n=3). Values indicated with * are significantly different from the negative control. *: P < 0.05, **: P<0.01, ***: P<0.001, ****: P<0.0001 (one-way ANOVA using Dunnett's multiple comparison test). (**B**) Multiple sequence alignment of selected cyanobacterial homologous CCRPs using MULTALIGN [1]. Alr0931, Slr1301 ($HmpF_{Syn}$; *Synechocystis*) and Synpcc7942_1139 ($HmpF_{Syc}$; *Synechococcus*) are identified by their designated cyanobase locus tag. Other proteins are given as NCBI accession numbers. WP_012412193.1 (*Nostoc punctiforme* PCC 73102), WP_009454477.1 (*Fischerella thermalis* PCC 7521), WP_016865706.1 (*Fischerella*), WP_016876359.1 (*C. fritschii* PCC 9212), WP_015199016.1 (*Calothrix* sp. PCC 6303), WP_009634512.1 (*Synechocystis* sp. PCC 7509), and WP_015954997.1 (*Cyanothece* sp. PCC 7424). Amino acids from 1-130 and 334-441 are depicted. Red highlighted amino acid residues are conserved among all listed species, blue amino acids are mostly conserved, and black amino acids are not conserved. Characteristic for this group of conserved cyanobacterial CCRPs is a highly conserved N-terminus with a M-L-Y-L-A-E-V sequence motif present in nearly all homologs, followed by a moderately conserved N-terminal region of the first 120 amino acids. Two other highly conserved domains are present in this group, one located around the centre of the proteins (between the 340th and 370th amino acid), and another one shortly thereafter between the 400th and 420th amino acid.

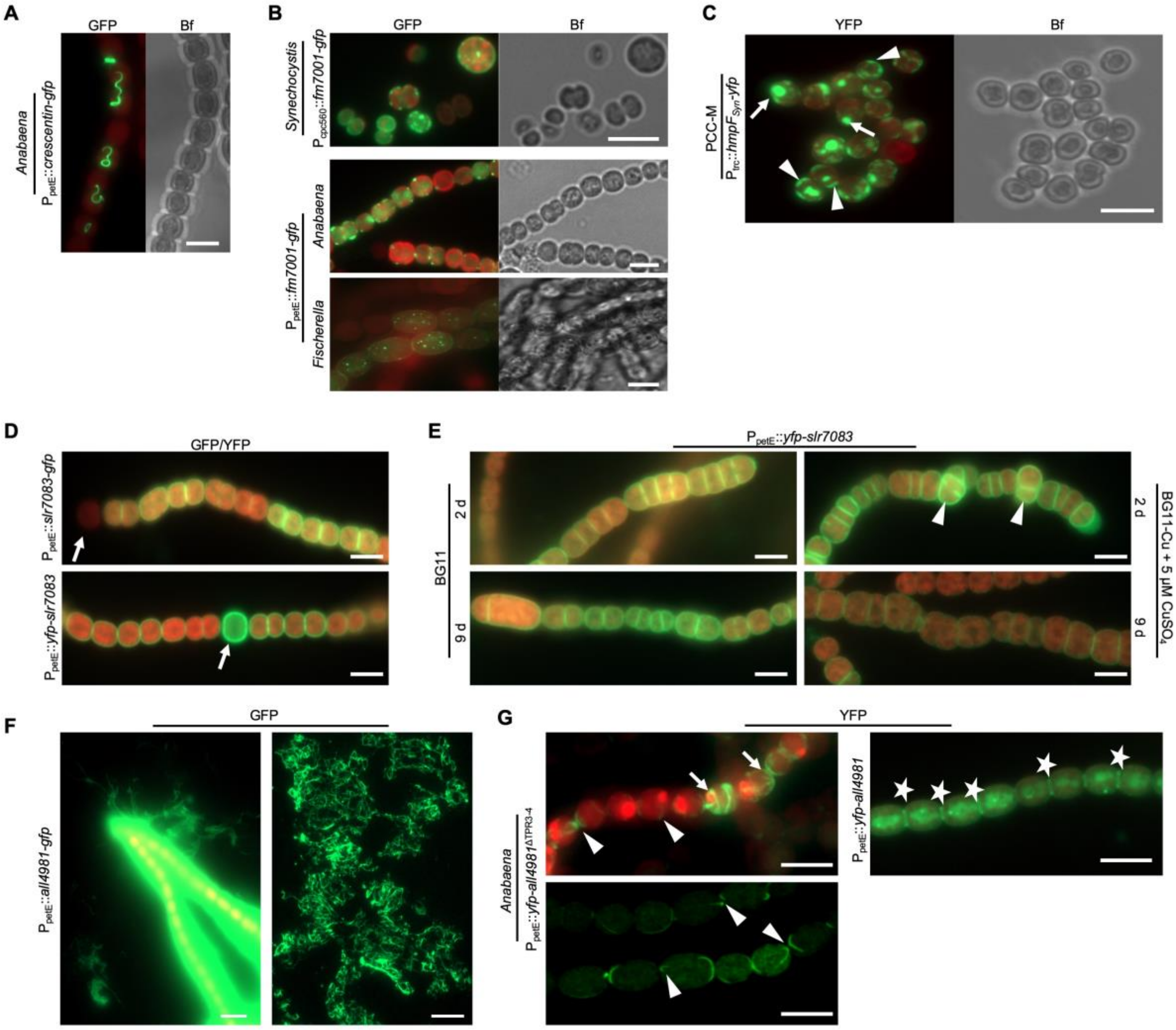

Supplementary Fig. 4: Expression of candidate CCRPs in different cyanobacterial species

GFP fluorescence, chlorophyll autofluorescence (red) and bright field micrographs of *Synechocystis*, PCC-M, *Anabaena* or *Fischerella* cells expressing (**A**) Crescentin-GFP, (**B**) Fm7001-GFP, (**C**) HmpF$_{Syn}$-YFP, (**D**) Slr7083-GFP, (**E**) YFP-Slr7083, (**F**) All4981-GFP or (**G**) YFP-All4981$_{\Delta TPR3-4}$ or YFP-All4981 from P$_{petE}$, P$_{trc}$, or P$_{cpc560}$. (**B**) Unlike N-terminal fusion with a YFP tag, no *in vivo* filament-like structures can be observed upon C-terminal fusion of Fm7001 with a GFP-tag in any tested cyanobacterium. (**C**) White triangles mark crescent-like localizations. White arrows show HmpF$_{Syn}$-YFP accumulations. (**D)** *Anabaena* cells were grown on BG11$_0$ plates. White arrows indicate heterocysts. (**E**) *Anabaena* cells expressing YFP-Slr7083 from P$_{petE}$ grown in liquid BG11 or liquid BG11 without copper and induced with 5 µM $CuSO_4$ for 2 and 9 d. White triangles point to multiseriate *Anabaena* trichome growth upon protein overexpression. (**F**) *Anabaena* cells expressing All4981-GFP from P$_{petE}$ grown in BG11 supplemented with 0.5 µM $CuSO_4$ for 2 d. Extended period of overexpression of All4981-GFP led to cell rupture. Protein released from the *Anabaena* trichome are shown in the left image while the right image shows extracellular (*ex vivo*) filament-like structures observed in the growth medium. (**G**) *Anabaena* cells were grown in BG11$_0$ supplemented with 0.5 µM $CuSO_4$. White triangles indicate selected filamentous strings traversing through the cells. White arrows point to spindle-like YFP-All4981$_{\Delta TPR3-4}$ structures. White stars mark septal localizations. Scale bars: (**A**-**D**,**G**) 5 µm, (**F**, left) 10 µm or (**F**, right) 20 µm.

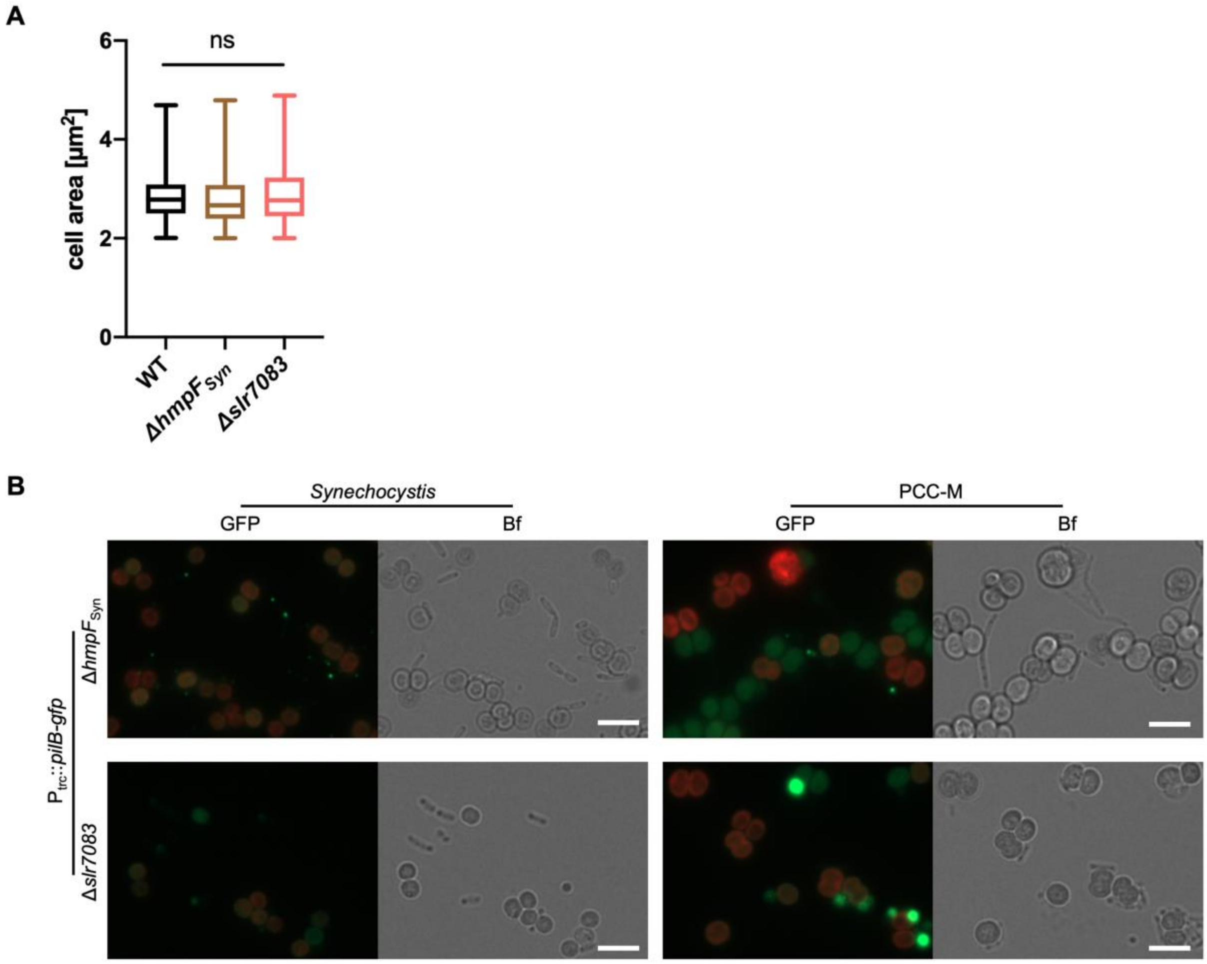

Supplementary Fig. 5: *Synechocystis* mutant phenotype and lack of PilB-GFP expression in *Synechocystis* and PCC-M mutant strains

(**A**) Measurement of cell area of *Synechocystis* WT, Δ*hmpF*$_{Syn}$ and Δ*slr7083* mutants as determined by microbeJ. (**B**) GFP fluorescence, chlorophyll autofluorescence (red) and bright field micrographs of *Synechocystis* or PCC-M Δ*hmpF*$_{Syn}$ or Δ*slr7083* mutant strains expressing PilB-GFP from P_{trc}. Cells were picked from mating filters directly as no exconjugant re-streaked on fresh selecting plates or transferred to liquid medium every survived, suggesting that these cells are possibly false-positives. Also, please note that the bright GFP spots in the micrographs are not from expression of PilB-GFP in the *Synechocystis* and PCC-M mutants but from expression of PilB-GFP in the *E. coli* used for mating that are still present on the mating filter. Scale bars: 5 µm.

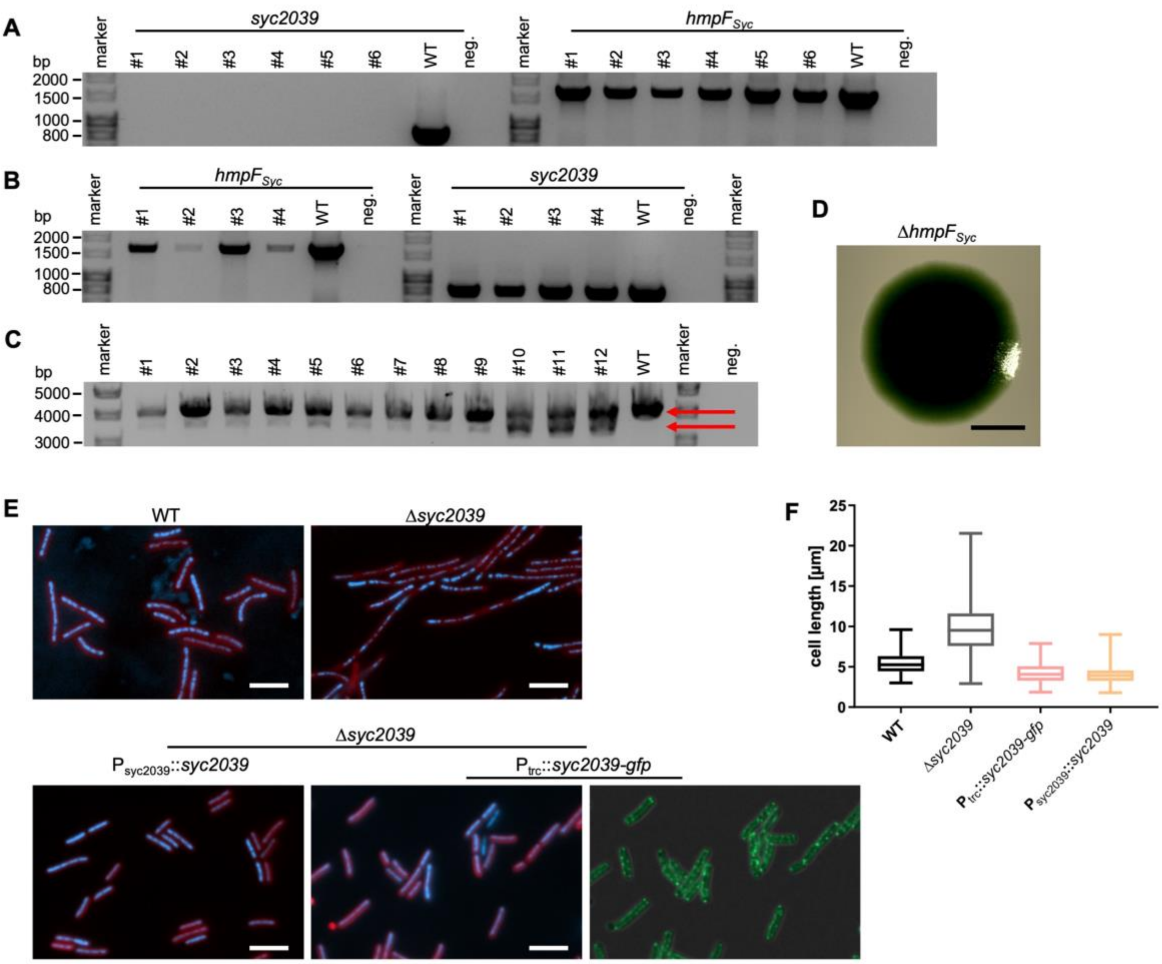

Supplementary Fig. 6: Verification of *Synechococcus* CCRP mutants

(**A**) Colony PCR of six Δ*syc2039* mutant clones using *syc2039* gene primers (#149/#147) and $hmpF_{Syc}$ gene primers (#161/#162) as a control. (**B**) Colony PCRs of four non-segregated Δ$hmpF_{Syc}$ mutant clones using $hmpF_{Syc}$ gene primers (#174/#175) or *syc2039 gene* primers (#159/#160) as a control. (**C**) Colony PCR of twelve non-segregated Δ$hmpF_{Syc}$ mutant clones using primers encompassing the homologous flanking regions used for homologous recombination (#238/#239). Upper red arrow indicates WT allele PCR product. Lower red arrow indicates Δ$hmpF_{Syc}$ mutant PCR product. As a positive control, *Synechococcus* genomic DNA was included. (**A**-**C**) Full gel pictures as shown in Extended Data Supplementary Fig. 2. (**D**) Growth of Δ$hmpF_{Syc}$ mutant on non-selective plates leads to a reversal to WT phenotype. (**E**) Merged DAPI fluorescence and chlorophyll autofluorescence (red) and merged GFP fluorescence and bright field micrographs of *Synechococcus* WT, Δ*syc2039* mutant and Δ*syc2039* mutant complemented with $P_{syc2039}$::*syc2039* or P_{trc}::*syc2039-gfp* inserted into the neutral NS1 locus. Cells were grown in BG11 or BG11 supplemented with 0.001 mM IPTG (for strain carrying P_{trc}::*syc2039-gfp*) and stained with 10 µg ml^{-1} DAPI. (**F**) Cell length of *Synechococcus* WT (n=505), Δ*syc2039* mutant (n=517), Δ*syc2039* mutant carrying P_{trc}::*syc2039-gfp* (n=547) and Δ*syc2039* mutant carrying $P_{syc2039}$::*syc2039* (n=529) cells.

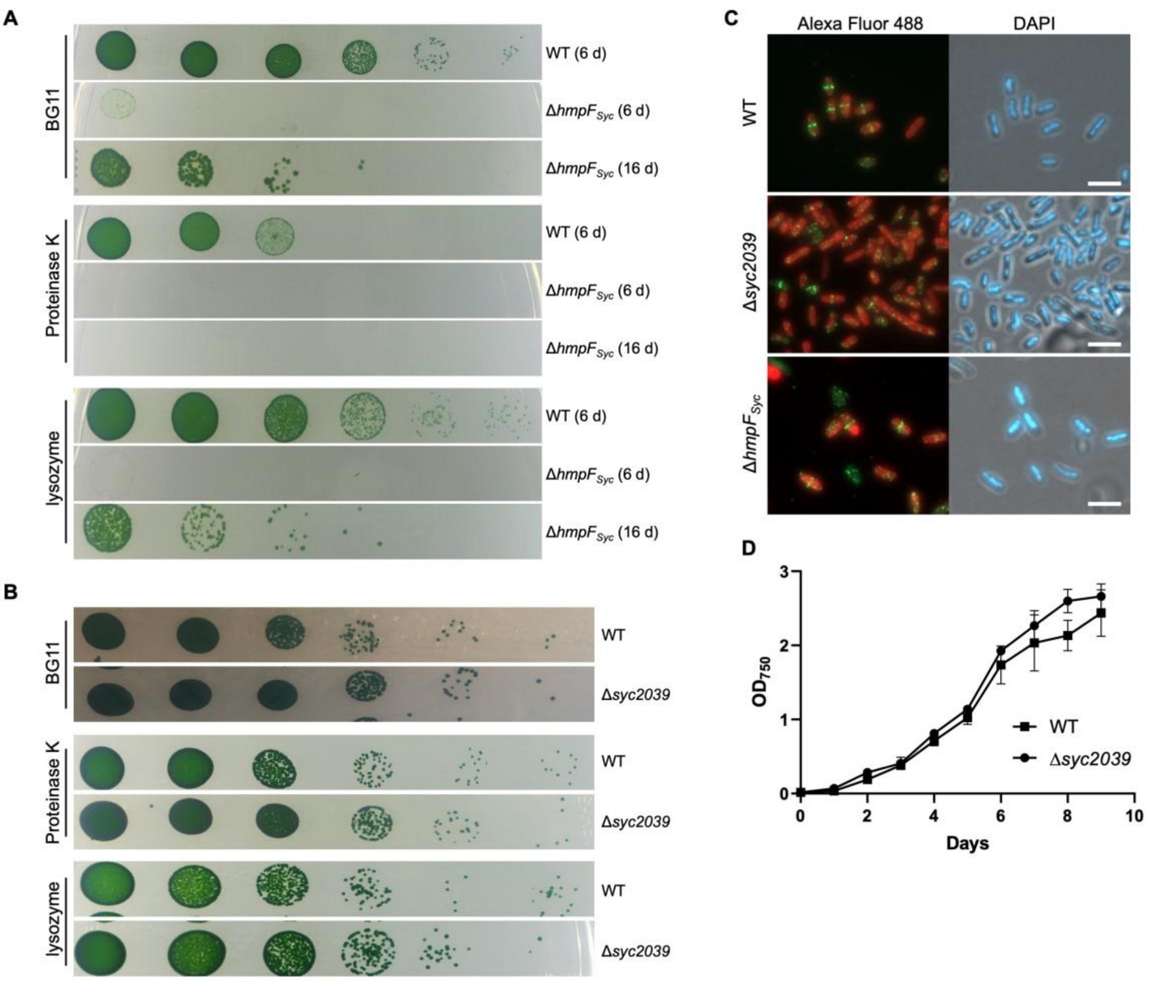

Supplementary Fig. 7: Phenotypic characterization of *Synechococcus* mutant strains

(**A**) *Synechococcus* WT (upper lane, after 6 days) and non-segregated Δ$hmpF_{Syc}$ mutant (middle lane: after 6 days and lower lane after 16 days) strains were grown on BG11 plates or BG11 plates supplemented with 50 µg ml^{-1} Km. Cells were resuspended in BG11, adjusted to an OD$_{750}$ of 0.4 and spotted in triplicates of serial 10-fold dilutions on BG11 plates or BG11 plates supplemented with 100 µg ml^{-1} Lysozyme or 50 µg ml^{-1} Proteinase K. Cells were grown until no further colonies arose in the highest dilution. (**B**) *Synechococcus* WT and Δ*syc2039* mutant strains were grown in liquid culture at standard growth conditions until an OD$_{750}$ of about 2.0, diluted in BG11 to an OD$_{750}$ of 0.4 and spotted in triplicates of serial 10-fold dilutions on BG11 plates or BG11 plates supplemented with 100 µg ml^{-1} Lysozyme or 50 µg ml^{-1} Proteinase K. Cells were grown until no further colonies arose in the highest dilution. (**C**) Merged Alexa Flour-488 fluorescence and chlorophyll autofluorescence (red) and merged bright field and DAPI fluorescence micrographs of *Synechococcus* WT, Δ*syc2039* or non-segregated Δ$hmpF_{Syc}$ mutant strains grown on BG11 plates and subjected to immunofluorescence staining using an anti-FtsZ primary antibody (Agrisera, raised against *Anabaena* FtsZ) and an Alexa Fluor-488 coated secondary antibody. Cells were mounted in Prolong Diamond antifade mountant with DAPI (Thermo Fischer Scientific). Scale bars: 5 µm. (**D**) Growth curve of *Synechocystis* WT and Δ*syc2039* mutant strain. Cells were grown in BG11, adjusted to an OD$_{750}$ of 0.1 and then grown in triplicates at standard growth conditions. OD$_{750}$ values were recorded once a day for 9 d. Error bars show the standard deviation (n=3).

Extended Data Supplementary

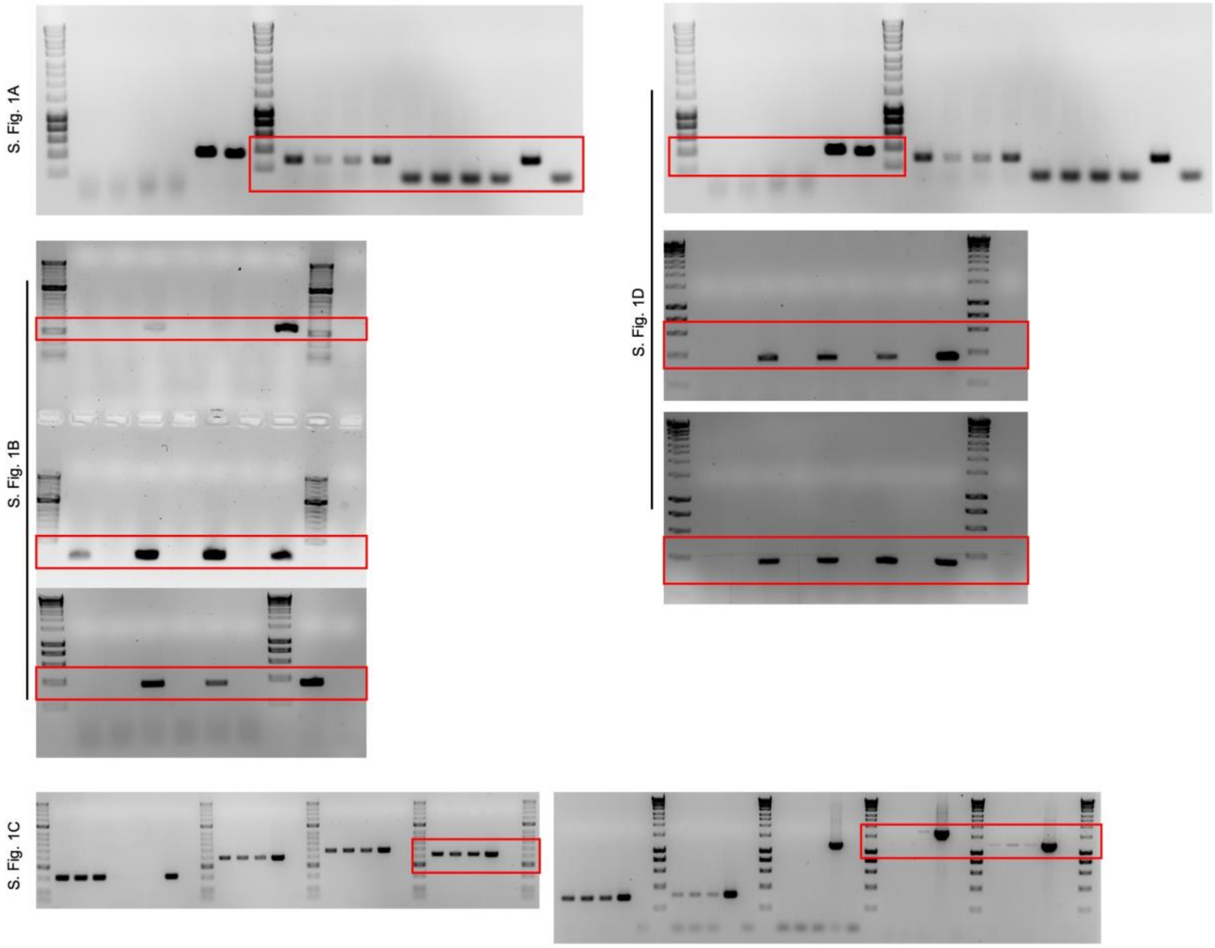

Extended Data Supplementary Fig. 1: Full images of gel pictures used in Supplementary Fig 1. Regions shown in the gel pictures of Supplementary Fig. 1 are indicated by red boxes.

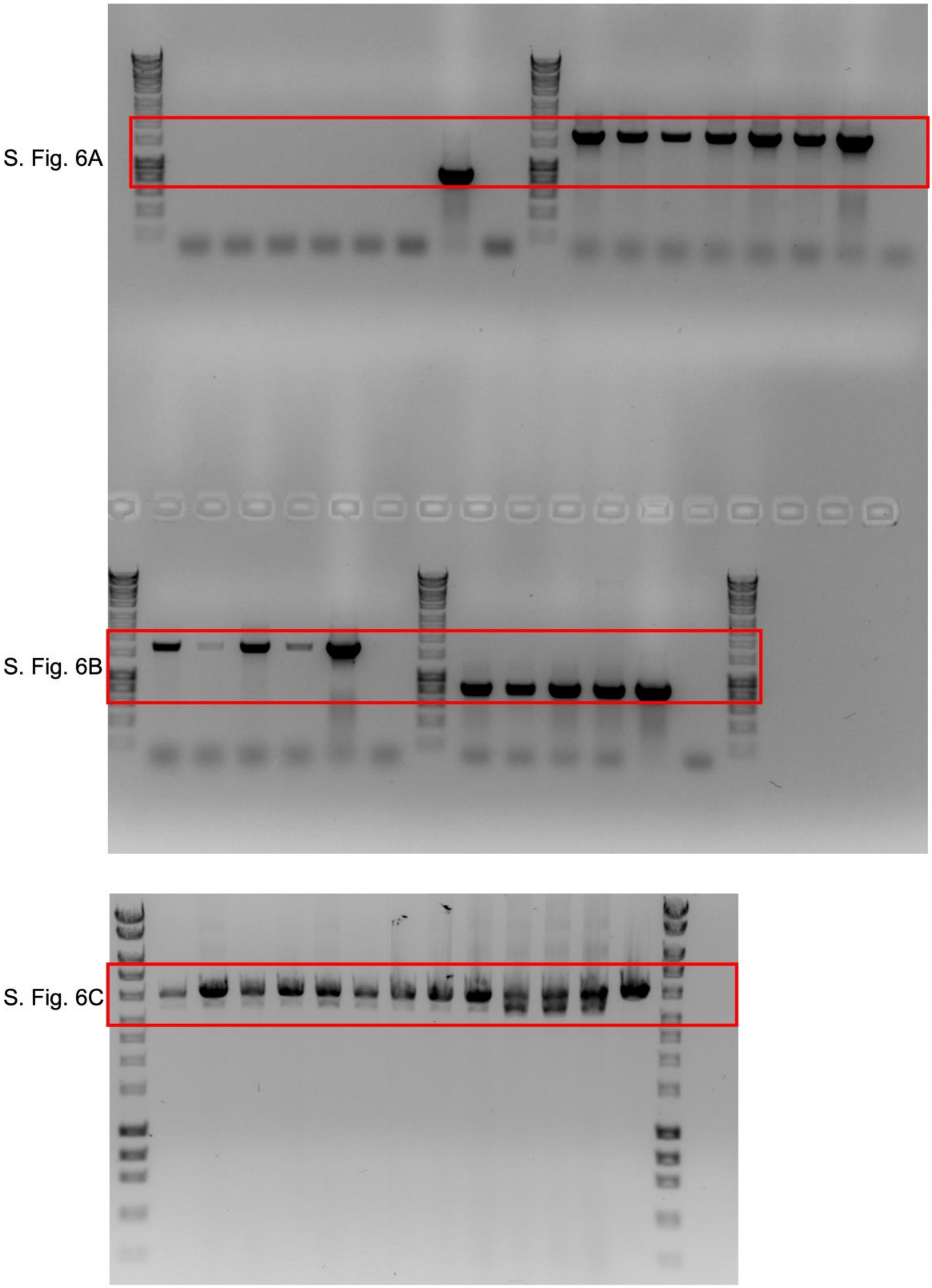

Extended Data Supplementary Fig. 2: Full images of gels shown in Supplementary Fig. 6

Regions shown in the gel pictures of Supplementary Fig. 6 are indicated by red boxes.

Supplementary Table 1: Properties of cyanobacterial CCRPs

Gene /Locus tag	Genus	Subsection	Homologs distribution	Predicted proteins of similar structure (I-TASSER)	Homolog similarities	Conserved domains	Others
crescentin	*C. crescentus*	n/a	n/a	Cytoplasmic domain of bacterial cell division protein EzrA		SMC, CCDC158	IF-like CCRP
filP	*S. coelicolor*	n/a	n/a	Dynein tail; α-Actinin; Tropomyosin		DUF3552, SMC, RNase_Y	IF-like CCRP
desmin	*Homo sapiens*	n/a	n/a	PI4KIIIa lipid kinase		Filament (pfam00038), SMC, Spc7, MscS_TM	IF protein
vimentin	*Homo sapiens*	n/a	n/a	PI4KIIIa lipid kinase		Filament (pfam00038), SMC, Spc7	IF protein
syc2039	*Synechococcus*	I	I	Tropomyosin		SMC, MukB, CALCOCO1	N-terminal TMD; only in *Synechococcus* sp.
syc1139	*Synechococcus*	I	I, II, III, IV, V	Cytoplasmic domain of bacterial cell division protein EzrA	39%	SMC, MukB, Spc7	Homolog to *slr1301* and HmpF
slr6096	*Synechocystis*	I	I, III, IV	Cytoplasmic domain of bacterial cell division protein EzrA		SMC	
slr7083	*Synechocystis*	I	I	Plectin		SMC, MscS_TM	Encoded on pSYSA plasmid, only in *Synechocystis* sp.
slr1301	*Synechocystis*	I	I, II, III, IV, V	Cytoplasmic domain of bacterial cell division protein EzrA	39%	SMC, SbcC, APG6, DUF3552	Homolog to *syc1139* and HmpF
tlr0420	*BP-1*	I	I, III	Plectin		SMC, MscS_TM	
fm7001	*Fischerella*	V	IV, V	α-catenin or vinculin; similarity to acyl-CoA dehydrogenase	63%	Acetyl-CoA carboxylase carboxyl transferase (PLN0322)	Highly expressed; 3' end 9 bp overlap to *fm7000*
fm6009	*Fischerella*	V	V	Structure of β-catenin and HTCF-4		COG0610	
all4981	*Anabaena*	IV	III, IV, V	TTC7B/Hyccin Complex, Clathrin	47%	TPR	5' with a 4 bp overlap to *all4982*

The first column indicates the respective gene name or locus tags of each protein candidate. The second and third column indicate the respective subsection of the corresponding cyanobacterial genus. Column four lists the subsections that contain homologous proteins to the respective CCRP. Column five indicates structural similarities of the candidate to proteins in the Protein Data Bank (PDB) based on I-TASSER[2,3]. The sixth column lists predicted sub-domains of protein candidates identified by BLAST Conserved Domain Search. Column seven names other features of interest. Abbreviations: (TMH) Transmembrane helix; (DUF) Domain of unknown function; (CCDC158) Coiled-coil domain-containing protein 158; (SMC) Structural maintenance of chromosomes; (MukB) The hinge domain of chromosome partition protein MukB; (APG6) Autophagy protein Apg6, (SbcC) DNA repair exonuclease SbcCD ATPase; (CALCOCO1) Calcium binding and coiled-coil domain; (Spc7) Spc7 kinetochore protein; (Filament) Intermediate filament protein; (TPR): Tetratricopeptide repeat; (PLN0322) Acetyl-CoA carboxylase carboxyl transferase; (COG0610) Type I site-specific restriction-modification system. n/a: not applicable. The employed identifiers correspond to respective RefSeq accession numbers listed in the brackets: Synpcc7942_2039 (ABB58069); Synpcc7942_1139 (ABB57169); Slr6096 (BAD02153.1); Slr7083 (BAD01985.1); Slr1301 (BAA16854.1); Tlr0420 (NP_681210.1); Fm7001 (WP_016868005.1); Fm6009 (WP_020476706); All4981 (BAB76680.1).

Supplementary Table 2: Bacterial strains

Strain	Genotype	Resistance	Reference
E. coli XL1 blue	*endA*1 *gyrA*96(nal$_R$) thi-1 *recA*1 *relA*1 *lac glnV*44 F'[::Tn10 *proAB*$_+$ *lacI*$_q$ Δ(*lacZ*)M15] *hsd*R17(r$_{K-}$ m$_{K+}$)	Tet	Stratagene
E. coli HB101	F$_-$ *mcrB mrr hsd*S20(r$_{B-}$ m$_{B-}$) *recA*13 *leuB*6 *ara*-14 *proA*2 *lacY*1 *galK*2 *xyl-5 mtl*-1 *rpsL*20(Sm$_R$) *gln*V44 λ$_-$	Sm	[4]
E. coli DH5α	F– Φ80*lac*ZΔM15 Δ(*lacZ*YA-*arg*F) U169 *recA*1 *endA*1 *hsdR17* (rK–, mK+) *phoA supE*44 λ– *thi*-1 *gyrA*96 *relA*1		[5]
E. coli DH5αMCR	F- *endA*1 *supE*44 *thi-1* λ$_-$ *recA1 gyrA*96 *relA*1 *deoR* Δ(*lacZYA-argF*)U169 Φ80d*lac*ZΔM15 *mcrA* Δ(*mrr hsdRMS mcrBC*)		[6]
E. coli BL21 (DE3)	F$_–$ *ompT gal dcm lon hsdS$_B$*(*r$_{B–}$m$_{B–}$*) λ(DE3 [*lacI lacUV5-T7p07 ind1 sam7 nin5*]) [*malB*$_+$]$_{K-12}$(λ$_S$)		[7]
E. coli BTH101	F$_–$, *cya-99*, *araD139*, *galE15*, *galK16*, *rpsL1* (*Str$_r$*), *hsdR2, mcrA*, *mcrB1*	Sm	Euromedex
Fischerella muscicola PCC 7414	WT		PCC, France
Synechocystis sp. PCC 6803	Glucose tolerant Kazusa substrain WT		PCC, France
BLS4	Δ*hmpF$_{Syn}$*::CS.3	Sm,Sp	This study
BLS5	Δ*slr7083*::CS.3	Sm,Sp	This study
Synechocystis sp. PCC-M 6803	Glucose tolerant and motile Moscow PCC-M substrain WT		A gift from Annegret Wilde (University Freiburg)
BLS6	Δ*hmpF$_{Syn}$*::CS.3	Sm,Sp	This study
BLS7	Δ*slr7083*::CS.3	Sm,Sp	This study
Synechococcus elongatus PCC 7942	WT		A gift from Martin Hagemann (University Rostock)
BLS8	Non-segregated Δ*hmpF$_{Syc}$*::*nptII*	Km	This study
BLS9	Δ*syc2039*::*nptII*	Km	This study
Anabaena sp. PCC 7120	WT		PCC, France

Supplementary Table 3: Oligonucleotides and Plasmids

#	Given name	Sequence 5' -> 3'	Purpose
1	Fm7001_intern_A	AGCGGGAAGATGGCTACTATC	RT-PCR
2	7001_northern	TCTGCGGCTTGACTTGATAC	RT-PCR
3	qftsZ7414_fwd	TGGAACTAAAGCTGCCGAGG	RT-PCR
4	qftsZ7414_rev	CTGTACCAGTTCCACCACCC	RT-PCR
5	Syn017_intern_A	TGCAACAGCAAACGGAACAG	RT-PCR
6	Syn017_intern_B	TTGGGAGCTAACTTGCCCAC	RT-PCR
7	rnpb 6803 primer fwd	GGAGTTGCGGATTCCTGTCA	RT-PCR
8	rnpb 6803 primer rev	AAGACCAACCTTTGCCCCTC	RT-PCR
9	Syn708_intern_A	TGGAGTGCCCTGCCTAACG	RT-PCR
10	Syn708_intern_B	CCCTTCTAACCTTTGTCGGGC	RT-PCR
11	Syc484_intern_A	CTACCATTCTTGGTGTGGCGG	RT-PCR
12	Syc484_intern_B	GAAATCCTGCGATCGCTGTTG	RT-PCR
13	Syc879_intern_A	CCTGTGACTTCTCTCCAGGG	RT-PCR
14	Syc879_intern_B	CTTTTAACTCGCGATCGCGGC	RT-PCR
15	Nos389_intern_A	ATCACCTGAATTAGCTGCGG	RT-PCR
16	Nos389_intern_B	CTAATAATGCCGCAATCAGCG	RT-PCR
17	All4982_intern_A	ATCAGATGGTGGAGGGAAGC	RT-PCR
18	All4983_intern_A	AGTAGCTGCATTTATCGGTGC	RT-PCR
19	pET19bmod-Fwd	GGAATTGTGAGCGGATAACAATT	Sequencing of pET21a(+) inserts
20	T7R	CTAATACGACTCACTATAGGGA	Sequencing of pET21a(+) inserts
21	pAM2991_Seq_A	GCGCCGACATCATAACGGTTC	Sequencing of pAM2991 inserts
22	pAM2991_Seq_B	GCTGAAAATCTTCTCTCATCCGCC	Sequencing of pAM2991 inserts
23	pRL153_Seq_Rev	AGGAGATTAACCCGCCCAAG	Sequencing of pRL153 inserts
24	CS3_Seq_Fwd	CGCGCAGATCAGTTGGAAG	Sequencing of gene replacement plasmids
25	CS3_Seq_Rev	AACGTCGGTTCGAGATGGC	Sequencing of gene replacement plasmids
26	GFP_Seq_Rev	TTGTGCCCATTAACATCACCATC	Sequencing of GFP containing plasmids
27	pJET1.2 forward sequencing primer	CGACTCACTATAGGGAGAGCGGC	Sequencing of pJET1.2 inserts
28	pJET1.2 reverse sequencing primer	AAGAACATCGATTTTCCATGGCAG	Sequencing of pJET1.2 inserts
29	pKO_Seq_Fwd	GCCTTTTTACGGTTCCTGGC	Sequencing pTHS121

30	pKO_Seq_Rev	TCTTTTCTACGGGGTCTGACG	Sequencing pTHS121
31	pIGA_Seq_Fwd	TGCGCATAGAAATTGCATCA	Sequencing of pIGA inserts
32	pIGA_Seq_Rev	GTCAGCAACACCTTCTTCA	Sequencing of pIGA inserts
33	pRL271_Seq_Fwd	GCCTGGTGCTACGCCTGAATA	Sequencing of pRL271 inserts
34	pRL271_Seq_Rev	CCAGTTAATAGTTTGCGCAACGTTG	Sequencing of pRL271 inserts
35	pRL278_Seq_Fwd	GGGGCGTAATTTTTTTAAGGCAGTTATTG	Sequencing of pRL278 inserts
36	pSL2680_Seq_A	CAAGAGGGCAAAAAACTCAATTTG	Sequencing of pSL2680 inserts
37	cpf1_1A	TTGGTCATGAGATTATCAAAAAGGATCCT GGAAAACGTTCTTCGGGGC	Amplification of *cpf1* for pTHS123
38	pRL25c_CRISPR_2 B	AGGCCCTTTCGTCTTCAAGAATTCTTTAC ACTGATGAATGTTCCGTTGCG	Amplification of *cpf1* for pTHS123
39	cpf1-1	CTCCAGAAGCTATAAACTATGAAC	Sequencing of *cpf1*
40	cpf1-2	CTACTTCAAGCTAGTGCGGAA	Sequencing of *cpf1*
41	cpf1-3	GTTGAAAATCAAGGCTACAAACTAAC	Sequencing of *cpf1*
42	cpf1-4	CGTTTCAAGGTAGAGAAGCAGG	Sequencing of *cpf1*
43	pRL25c_Seq_Fwd	CTTTGATCTTTTCTACGGGGTCT	Sequencing of pRL25C inserts
44	pRL25c_Seq_Rev	TTGAGGTGAGGGATGAGCG	Sequencing of pRL25C inserts
45	pMAL_Seq_Fwd	AGAAAGGTGAAATCATGCCG	Sequencing of pMAL-c2x inserts
46	pMAL_Seq_Rev	CTGCAAGGCGATTAAGTTGG	Sequencing of pMAL-c2x inserts
47	MB_Seq_A	GGCTCGTATGTTGTGTGG	Sequencing of pKNT25, pKT25 and pUT18 inserts
48	MB_Seq_B	GGCTTAACTATGCGGCATC	Sequencing of pKNT25, pUT18 and pUT18C inserts
49	MB_Seq_C	TAACGCCAGGGTTTTCCCA	Sequencing of pKT25 inserts
50	pKNT25_Seq_Rev	CGTTTGCGTAACCAGCC	Sequencing of pKNT25 inserts
51	pKT25_Seq_Fwd	GATTCGGTGACCGATTACCTG	Sequencing of pKT25 inserts
52	pUT18_Seq_Rev	GATGCGTTCGCGATCCAG	Sequencing of pUT18 inserts

53	pUT18C_Seq_Fwd	TCGCCGGATGTACTGGAAAC	Sequencing of pUT18C inserts
54	N-term_1A	GAGGATCCCCGGGTACC	Amplification of pKNT25 and pUT18
55	N-term_1B	TAGAGTCGACCTGCAGGCA	Amplification of pKNT25 and pUT18
56	pKT25_1A	CCCCGGGTACCTAAGTAAGTAAG	Amplification of pKT25
57	pKT25_1B	ATCCTCTAGAGTCGACCCTGC	Amplification of pKT25
58	pUT18C_1A	CCGAGCTCGAATTCATCGAT	Amplification of pUT18C
59	pUT18C_1B	TACCCGGGGATCCTCTAGAGT	Amplification of pUT18C
60	pET21a_1A	CACCACCACCACCACCAC	Amplification of pET21a(+) for gfp fusions
61	pET21a_1B	ATGTATATCTCCTTCTTAAAGTTAAACAAAATTATTTCTAGAGG	Amplification of pET21a(+) for gfp fusions
62	pRL271_Fwd	GAGCTCGCGAAAGCTTGCATG	Amplification of pRL271 and pRL278
63	pRL271_Rev	CTCGAGATCTAGATATCGAATTTCTGCCAT	Amplification of pRL271
64	pRL278_Rev	CCGCTTATTATCACTTATTCAGGCG	Amplification of pRL278
65	pETM22_Vec_R	ATGTTTTTCGTATTTTCCCTACCAGAAGAATGATGATGATGATGG	Amplificaiton of pETM22
66	pETM22_Vec_F	TGGATGAACTATACAAATAAATCCGGCTGCTAACAAAGC	Amplificaiton of pETM22
67	Vector.FOR	TGATGTTCAACTTCGACAGCGAATTCCTCGACCTGCAGGG	Amplification of pIGA
68	Vector.REV	AGGGACTCTTCTCTACAGGTGGTACCCCGGGTTCGAAATCG	Amplification of pIGA
69	YFP_pcpc560_2A	CATAAAGTCAAGTAGGAGATTAATTCAATGCTGAGCAAGGGCGA	Amplification of YFP with overhang to P_{cpc560}
70	YFP_2A	TACAGGTTAGGAGAACGCCATGCTGAGCAAGGGCG	Amplification of YFP
71	YFP-Myc_2B	CAGATCCTCTTCAGAGATGAGTTTCTGCTCCTTGTACAGCTCGTCCATGC	Amplification of YFP with C-terminal *myc*
72	Myc+Linker_2B	TCCTGAACCCGATCCAGAGCCCAGATCCTCTTCAGAGATGAGTTTC	Amplification of YFP with C-terminal *myc* and a GSGSGSG linker

73	XIII-GFP_1A	GCTAGCGACTCGACCGGTTC	Amplification of pRL153
74	XIII-GFP_1B	GCTTAATTTCTCCTCTTTAATTCTAGGTACCCG	Amplification of pRL153
75	eYFP_MYC_3A	GAGCAGAAACTCATCTCTGAAGAGGATCTGATGCTGAGCAAGGGCGAG	*yfp* for pTHS82
76	eYFP_3B	AACCGGTCGAGTCGCTAGCTTACTTGTACAGCTCGTCCATGC	*yfp* for pTHS82
77	GFP_pAM2991_2B	GCCTGCAGGTCGACTCTAGATTATTTGTATAGTTCATCCATGCCATGTG	$hmpF_{Syc}$-*gfp* for pTHS74
78	His_pAM2991_2B	GCCTGCAGGTCGACTCTAGATCAGTGGTGGTGGTGGTG	*syc2039-gfp-his* for pTHS75
79	CS.3_Fwd	GATCCGTGCACAGCACCTTG	Amplification of CS.3 cassette
80	CS.3_Rev	TTATTTGCCGACTACCTTGGTGATCT	Amplification of CS.3 cassette
81	nptII_Fwd	ATGATTGAACAAGATGGATTGCACG	Amplification of nptII
82	nptII_Rev	TCAGAAGAACTCGTCAAGAAGGCG	Amplification of nptII
83	pUC_ori_Fwd	TTGAGATCCTTTTTTTCTGCGCGTAATC	Amplification of pUC origin
84	pUC_ori_Rev	TTTCCATAGGCTCCGCCCC	Amplification of pUC origin
85	nptII_A	AAGCTTCACGCTGCCGCAA	Amplification of P_{nptII}::*nptII*
86	nptII_B	TCAGAAGAACTCGTCAAGAAGGCG	Amplification of P_{nptII}::*nptII*
87	GFP_pET21a_2B	AGTGGTGGTGGTGGTGGTGTTTGTATAGTTCATCCATGCCATGTGTAATC	Amplification of *gfp*-fusions for insertion into pET21a(+)
88	7001_EcoRI_Fwd	GCATGAATTCAGGGAAAATACGAAAAACATTG	*fm7001* for pTHS73
89	7001_His_PstI_R	GCTACTGCAGTCAGTGGTGATGGTGATGATGGACTAAGGCAGTCATTAAATAGT	*fm7001* for pTHS73
90	Fragment 1.FOR	GATTTCGAACCCGGGGTACCACCTGTAGAGAAGAGTCCCTGAATATCAA	P_{cpc560} for pTHS60
91	Fragment 1.REV	TTTTTCGTATTTTCCCTCATTGAATTAATCTCCTACTTGACTTTATGAGTTGGGA	P_{cpc560} for pTHS60
92	Fragment 2.FOR	TCAAGTAGGAGATTAATTCAATGAGGGAAAATACGAAAAACATTGGAGT	*fm7001* for pTHS60
93	Fragment 2.REV	GCACTAGCAGATGCACTAGCGACTAAGGCAGTCATTAAATAGTGAAGTAAGTAATTAAAGTAAC	*fm7001* for pTHS60
94	Fragment 3.FOR	ATTTAATGACTGCCTTAGTCGCTAGTGCATCTGCTAGTGCTAGT	*gfp*-tagged genes for pTHS60
95	Fragment 3.REV	CCGACAATCCAAACACCGGTTTATTTGTATAGTTCATCCATGCCATGTGTAATCC	*gfp*-tagged genes for pTHS60

96	Fragment 4.FOR	TGGATGAACTATACAAATAAACCGGTGTTTGGATTGTCGG	T_{rbcL} for pTHS60
97	Fragment 4.REV	CCCTGCAGGTCGAGGAATTCGCTGTCGAAGTTGAACATCAGTAAGC	T_{rbcL} for pTHS60
98	TrbcL_A	ACCGGTGTTTGGATTGTCGG	Amplification of pIGA containing P_{cpc560} and T_{rbcL} for pTH76
99	pIGA_Pcpc560_1B	TGAATTAATCTCCTACTTGACTTTATGAGTTGGG	Amplification of pIGA containing P_{cpc560} and T_{rbcL} for pTH76
100	YFP_pcpc560_2A	CATAAAGTCAAGTAGGAGATTAATTCAATGCTGAGCAAGGGCGA	*yfp-fm7001* for pTHS76
101	7001_TrbcL_2B	CCGACAATCCAAACACCGGTTCAGACTAAGGCAGTCATTAAATAGTGAAG	*yfp-fm7001* for pTHS76
102	7001F_BamHI	ACTGGATCCAGGGAAAATACGAAAAACATTGGA	*fm7001* for pTHS63
103	7001R_NotI	AGCGGCCGCTCAGACTAAGGCAGTCATTAAATAGTG	*fm7001* for pTHS63
104	7001F_BamHI	GGATCCTATGAGGGAAAATACGAAAAAC	*fm7001* for pTHS95, pTHS96, pTHS97 and pTHS98
105	7001R_EcoRI	AGCGAATTCTCAGACTAAGGCAGTCAT	*fm7001* for pTHS96 and pTHS98
106	7001R_SacI	GAGCTCCTGACTAAGGCAGTCATTA	*fm7001* for pTHS95 and pTHS97
107	7120petER_7001ol	GTATTTTCCCTCATACCTGTAGTTTTATTTTTCTTATTTC	*petE* for pTHS83 (overlap PCR)
108	7001F_petEol	AAAACTACAGGTATGAGGGAAAATACGAAAAAC	*fm7001* for pTHS83 (overlap PCR)
109	7001R_SacI_C	AGCGAGCTCTAAGGCAGTCATTAAATAGTG	*fm7001* for pTHS83
110	7001F_NheI	AACTGCTAGCAGGGAAAATACGAAAAAC	*fm7001* for pTHS83
111	7001_3A	CTCTGGATCGGGTTCAGGAATGAGGGAAAATACGAAAAACATTGG	*fm7001* for pTHS84
112	7001_3B	CCTTTCGTCTTCAAGAATTCTTCAGACTAAGGCAGTCATTAAATAGTG	*fm7001* for pTHS84
113	pRL271_7up_F	AGAAATTCGATATCTAGATCTCGAGAGCAATGTGAGTGAGTTCGTGAGC	Upstream homology for pTHS126
114	7001KO_1B	GTGCTTGCGGCAGCGTGAAGCTTGGGGTTATCCTTAATAGAAGAAGAGTGC	Upstream homology for pTHS126
115	7001KO_2A	CGCCTTCTTGACGAGTTCTTCTGAATCAAGAGCATTCTTGATTTCTGTCTCA	Downstream homology for pTHS126

116	pRL271_7down_R	CAGGCATGCAAGCTTTCGCGAGCTCTGCTACCAAGACGATGCGTTTCATGTC	Downstream homology for pTHS126
117	7001KO_2A2	AATTCGATATCTAGATCTCGAGTTGCGTTTCAAAACACTACAAATTAGTACAAAC	Upstream homology for pTHS127
118	7001KO_2B2	AAGGTGCTGTGCACGGATCGGGGTTATCCTTAATAGAAGAAGAGTGC	Upstream homology for pTHS127
119	7001KO_4A2	CAAGGTAGTCGGCAAATAAATCAAGAGCATTCTTGATTTCTGTCTC	Downstream homology for pTHS127
120	7001KO_4B2	TGCAAGCTTTCGCGAGCTCCTGAAGACAAAGATGAAGTTTCGATATTACC	Downstream homology for pTHS127
121	trunc7001_2A	CTGAATAAGTGATAATAAGCGGTTGCGTTTCAAAACACTACAAATTAGTACAAA	Truncated *fm7001* for pTHS128
122	trunc7001_2B	TGCAAGCTTTCGCGAGCTCGTATTGATACTGGGTTGAGAATACTGC	Truncated *fm7001* for pTHS128
123	7001_gRNA_A	AGATGAGTTTTGCACAAAGTTGGA	*fm7001* gRNA for pTHS121 and pTHS123
124	7001_gRNA_B	AGACTCCAACTTTGTGCAAAACTC	*fm7001* gRNA for pTHS121 and pTHS123
125	Fm7001_HL1A	TTGTCTAGCTTTAATGCGGTAGTTGGTACCAGGAACATCGCGTCTCTACC	Downstream homology repair template for pTHS121 and pTHS123
126	Fm7001_HL1B	AAGAATGCTCTTGATGGGGTTATCCTTAATAGAAGAAGAGTGC	Upstream homology repair template for pTHS121 and pTHS123
127	Fm7001_HL2A	TATTAAGGATAACCCCATCAAGAGCATTCTTGATTTCTGTCTC	Upstream homology repair template for pTHS121 and pTHS123
128	Fm7001_HL2B	GATTACAGATCCTCTAGAGTCGACGGTACCTAAGGCAGCAACGTTTTCCG	Downstream homology repair template for pTHS121 and pTHS123
129	Syn017_NdeI_fwd	GCTACATATGACAAGTCAAAATTTTGTTTCTGAT	*slr7083* for pTHS61
130	Syn017_XhoI_wo_rev	GCTACTCGAGTGGTAAATAAGGGGGAGTGG	*slr7083* for pTHS61
131	pIGA_V_017_R	ACAAAATTTTGACTTGTCATTGAATTAATCTCCTACTTGACTTTATGAGTTGG	Amplification of pIGA with P_{cpc560} and T_{rbcL} for pTHS77

132	pIGA_V_017_F	CCACTCCCCCTTATTTACCAGCTAGTGCATCTGCTAGTGCT	Amplification of pIGA with P_{cpc560} and T_{rbcL} for pTHS77
133	pIGA_Syn017_F	TCAAGTAGGAGATTAATTCAATGACAAGTCAAAATTTTGTTTCTGATCAAG	*slr7083* for pTHS77
134	pIGA_Syn017_R	GCACTAGCAGATGCACTAGCTGGTAAATAAGGGGGAGTGGGAC	*slr7083* for pTHS77
135	Syn017_pRL25c_F	TAAAACTACAGGTTAGGAGAACGCCATGACAAGTCAAAATTTTGTTTCTGATCAAGATACC	*slr7083* for pTHS86
136	Syn017_pRL25c_R	CACTAGCACTAGCAGATGCACTAGCTGGTAAATAAGGGGGAGTGGGACG	*slr7083* for pTHS86
137	017_up_Fwd	GTCAGGGGGGCGGAGCCTATGGAAATTTCCCAACAAATTAGTGAATGGCAGG	Upstream homology for pTHS120
138	017_up_Rev	CGTGCAATCCATCTTGTTCAATCATTGTTTTACCAGATTAAAATTAATGATTGAGACAGAATTGAAAAG	Upstream homology for pTHS120
139	017_down_Fwd	TCGCCTTCTTGACGAGTTCTTCTGAGATTTCCAGTCACTTCCGTTTTTTTACTATTAGG	Downstream homology for pTHS120
140	017_down_Rev	TACGCGCAGAAAAAAAGGATCTCAAAATCCTAAAATTCGTGGTTCATCTTTCTTTGT	Downstream homology for pTHS120
141	Syn017_3A	CTCTGGATCGGGTTCAGGAATGACAAGTCAAAATTTTGTTTCTGATC	*slr7083* for pTHS85
142	Syn017_3B	CCTTTCGTCTTCAAGAATTCTCTATGGTAAATAAGGGGGAGTGGG	*slr7083* for pTHS85
143	Syn017_pET_2A	GTTTAACTTTAAGAAGGAGATATACATATGACAAGTCAAAATTTTGTTTCTGATCAAG	*slr7083* for pTHS69
144	017_Seq_A	CCACAGCGGAAACCTTTTTAGATC	Sequencing of Δ*slr7083* plasmids
145	017_Seq_B	AAACGAACAGAGTGGAACTTTGC	Sequencing of Δ*slr7083* plasmids
146	Syc484_2A	GTTTAACTTTAAGAAGGAGATATACATATGAACTACGCTCTTACCCAAG	*syc2039* for pTHS87
147	Syc484_2B	AGTGGTGGTGGTGGTGGTGAGACCCTAACCAGCGGC	*syc2039* for pTHS87
148	Syc484_pIGA_2A	TAAAGTCAAGTAGGAGATTAATTCAATGAACTACGCTCTTACCCAAGC	*syc2039* for pTHS80
149	Syc484_2A2	TACAGGTTAGGAGAACGCCATGAACTACGCTCTTACCCAAG	*syc2039* for pTHS62
150	Syc484_2B2	CACTAGCAGATGCACTAGCAGACCCTAACCAGCGGC	*syc2039* for pTHS62
151	Syc484_pAM2991_2A	CACACAGGAAACAGACCATGAACTACGCTCTTACCCAAGC	*syc2039* for pTHS75
152	Syc484_KO_2A	TGTAGGAGATCTTCTAGAAAGATGTAGGCATCAAAGGCAGGC	Upstream homology for pTHS119

153	Syc484_KO_2B	CTTGCGGCAGCGTGAAGCTTAGCAAAGCAAAAGAAGCGATCG	Upstream homology for pTHS119
154	Syc484_KO_4A	TTCTTGACGAGTTCTTCTGATTCTGCTGCGATGCGTTAGG	Downstream homology for pTHS119
155	Syc484_KO_4B	CTCGAGTTTTTCAGCAAGATCAAGTAAGACTGGCTGCCATG	Downstream homology for pTHS119
156	Syc484_Seq_A	GATGCCACCGAGCAGAATTAG	Verification of Δ*syc2039*
157	Syc484_Seq_B	GGCAGATCAATCAGCAGCTC	Verification of Δ*syc2039*
158	Syc879_pET_2A	GTTTAACTTTAAGAAGGAGATATACATATGCTCTATCTGGCTGAAGTCG	*hmpF*$_{Syc}$ for pTHS66
159	Syc879_pET_2B	CAGTGGTGGTGGTGGTGGTGGGCTGCAATCAGTTGATGACT	*hmpF*$_{Syc}$ for pTHS66
160	Syc879_pIGA_2A	TAAAGTCAAGTAGGAGATTAATTCAGTGCTCTATCTGGCTGAAGTCGG	*hmpF*$_{Syc}$ for pTHS81
161	Syc879_2A	TACAGGTTAGGAGAACGCCATGCTCTATCTGGCTGAAGTCG	*hmpF*$_{Syc}$ for pTHS93
162	Syc879_2B	CACTAGCAGATGCACTAGCGGCTGCAATCAGTTGATGACTG	*hmpF*$_{Syc}$ for pTHS93
163	Syc879_pAM2991_2A	CACACAGGAAACAGACCATGCTCTATCTGGCTGAAGTCGG	*hmpF*$_{Syc}$ for pTHS74
164	Syc879_KO_2A	TGTAGGAGATCTTCTAGAAAGATCTGGAGCGATCGCTATGG	Upstream homology for pTHS133
165	Syc879_KO_2B	CTTGCGGCAGCGTGAAGCTTTATCGATGCCTCGCCTTAATCAATC	Upstream homology for pTHS133
166	Syc879_KO_4A	CCTTCTTGACGAGTTCTTCTGAGCCAGTCCCCCGCGACTA	Downstream homology for pTHS133
167	Syc879_KO_4B	CTCGAGTTTTTCAGCAAGATGGCAAGCGCAACTGAATTCTTAC	Downstream homology for pTHS133
168	Syn708_NdeI_F	GCTACATATGCTCTATCTGGCTGAAATTAAGAAA	*slr1301* for pTHS65
169	Syn708_XhoI_R_w/o	GCTACTCGAGACCGCCAAACAATAGGGTC	*slr1301* for pTHS65
170	Syn708_pET_2A	GTTTAACTTTAAGAAGGAGATATACATATGCTCTATCTGGCTGAAATTAAGAAAC	*slr1301* for pTHS67
171	Syn708_pRL25c_Fwd	TAAAACTACAGGTTAGGAGAACGCCATGCTCTATCTGGCTGAAATTAAGAAACAAAC	*slr1301* for pTHS91
172	Syn708_pRL25c_Rev	CACTAGCACTAGCAGATGCACTAGCACCGCCAAACAATAGGGTCT	*slr1301* for pTHS91
173	Syn708_3A	CTCTGGATCGGGTTCAGGAGTGCTCTATCTGGCTGAAATTAAG	*slr1301* for pTHS92
174	Syn708_3B	CCTTTCGTCTTCAAGAATTCTCTAACCGCCAAACAATAGGGTC	*slr1301* for pTHS92

175	153Syn708_2A	AGAATTAAAGAGGAGAAATTAAGCATGCT CTATCTGGCTGAAATTAAGAAAC	*slr1301* for pTHS82
176	153Syn708_2B	TCAGAGATGAGTTTCTGCTCACCGCCAAA CAATAGGGTC	*slr1301* for pTHS82
177	pJET708_2A	TGTAGGAGATCTTCTAGAAAGATAATAGA CTGCAATGTCAAAAAACTCAG	Upstream homology for pTHS131
178	708KO_CS3_2B	CAAGGTGCTGTGCACGGATCAAGTCGTT GTCCTGAGCAG	Upstream homology for pTHS131
179	708KO_CS3_4A	CCAAGGTAGTCGGCAAATAATTGGGTTG GTTGCCGAC	Downstream homology for pTHS131
180	pJET708_4B	CTCGAGTTTTTCAGCAAGATTTAGCAAGG TGGGGGGAATG	Downstream homology for pTHS131
181	708KO_1Aa	CCTGATTCTGTGGATAACCGTACGTCAAA ATCGAATTCCCGGTC	Upstream homology for pTHS132
182	708KO_1B	TGCTTGCGGCAGCGTGAAGCTTAAGTCG TTGTCCTGAGCAGTG	Upstream homology for pTHS132
183	708KO_2A	CGCCTTCTTGACGAGTTCTTCTGATTGGG TTGGTTGCCGACTTC	Downstream homology for pTHS132
184	708KO_2B	GATTATCAAAAAGGATCTTCACCTTTAGC AAGGTGGGGGGAATGC	Downstream homology for pTHS132
185	Nos389_pET_2A	GTTTAACTTTAAGAAGGAGATATACATAT GAATAGTGAGTTGTTCCAGAAGC	*all4981* for pTHS72
186	Nos389_NdeI_F	GCTACATATGAATAGTGAGTTGTTCCAGA AG	*all4981* for pTHS64
187	Nos389_XhoI_wo_R	GCTACTCGAGGATGTTACTATCACTACTT TGAATTTTT	*all4981* for pTHS64
188	Nos389_2A	CTACAGGTTAGGAGAACGCCATGAATAGT GAGTTGTTCCAGAAGCTAGC	*all4981* for pTHS89
189	Nos389_2B	GCACTAGCAGATGCACTAGCGATGTTACT ATCACTACTTTGAATTTTTTTGAGTTTGGC	*all4981* for pTHS89
190	Nos389_3A	CTCTGGATCGGGTTCAGGAATGAATAGT GAGTTGTTCCAGAAGC	*all4981* for pTHS88
191	Nos389_3B	CCTTTCGTCTTCAAGAATTCTTTAGATGTT ACTATCACTACTTTGAATTTTTTTGAG	*all4981* for pTHS88
192	Nos389_pIGA_2A	TAAAGTCAAGTAGGAGATTAATTCAATGA ATAGTGAGTTGTTCCAGAAGC	*all4981* for pTHS79
193	Nos389_TrbcL_2B	CCGACAATCCAAACACCGGTTTAGATGTT ACTATCACTACTTTGAATTTTTTTGAGTTT G	*all4981* for pTHS78
194	389KO_2A	ATTCGATATCTAGATCTCGAGTGTCAGAT TTAGTACTTTAAATACAAGACTTACACAC	Upstream homology for pTHS129

195	389KO_2B	CAAGGTGCTGTGCACGGATCAGCTGTTC GCTCTTGAGGG	Upstream homology for pTHS129
196	389KO_4A	CCAAGGTAGTCGGCAAATAAAGTAACGC GATGTGCGACT	Downstream homology for pTHS129
197	389KO_4B	ATGCAAGCTTTCGCGAGCTCGATTAATAC CTTTGGTGTTCATGACACTGG	Downstream homology for pTHS129
198	trunc389_2A	CTGAATAAGTGATAATAAGCGGAAGCCAT TTTAGATCGAGAGGCG	Truncated *all4981* for pTHS130
199	trunc389_2B	TGCAAGCTTTCGCGAGCTCGCTAAATTCC AAAACTCACTGCCTT	Truncated *all4981* for pTHS130
200	Nos389_gRNA-A	AGATCAGAAGCTAGCAAAAGCACA	*all4981* gRNA for pTHS124 and pTHS125
201	Nos389_gRNA-B	AGACTGTGCTTTTGCTAGCTTCTG	*all4981* gRNA for pTHS124 and pTHS125
202	Nos389_HL1A	TTTGTCTAGCTTTAATGCGGTAGTTGGTA CCGTGTGGGGTAATTTGCGGG	Upstream homology for pTHS124 and pTHS125
203	Nos389_HL1B	ATAAGTCGCACATCGCGTTACTTCATAGC TGTTCGCTCTTGAGG	Upstream homology for pTHS124 and pTHS125
204	Nos389_HR2A	CCTCAAGAGCGAACAGCTATGAAGTAAC GCGATGTGCGACTTATTC	Downstream homology for pTHS124 and pTHS125
205	Nos389_HR2B	GGATTACAGATCCTCTAGAGTCGACGGTA CCGGACACCACCAGCCATTTC	Downstream homology for pTHS124 and pTHS125
206	MB_1A	TGCCTGCAGGTCGACTCTAATGAATAGTG AGTTGTTCCAGAAGC	*all4981* for pTHS107 and pTHS109
207	MB_1B	TCGGTACCCGGGGATCCTCGATGTTACT ATCACTACTTTGAATTTTTTTGAGT	*All4981* for pTHS107 and pTHS109
208	MB_2A	AGGGTCGACTCTAGAGGATATGAATAGT GAGTTGTTCCAGAAGC	*all4981* for pTHS108
209	MB_2B	CTTACTTAGGTACCCGGGGGATGTTACTA TCACTACTTTGAATTTTTTTGAGT	*all4981* for pTHS108
210	MB_4A	TCTAGAGGATCCCCGGGTAATGAATAGT GAGTTGTTCCAGAAGC	*all4981* for pTHS110
211	MB_4B	TCGATGAATTCGAGCTCGGGATGTTACTA TCACTACTTTGAATTTTTTTGAGT	*all4981* for pTHS110
212	MB_9A	TGCCTGCAGGTCGACTCTAATGCTCTATC TGGCTGAAATTAAGAAAC	*slr1301* for pTHS111 and pTHS113

213	MB_9B	TCGGTACCCGGGGATCCTCACCGCCAAACAATAGGGT	*slr1301* for pTHS111 and pTHS113
214	MB_10A	AGGGTCGACTCTAGAGGATATGCTCTATCTGGCTGAAATTAAGAAAC	*slr1301* for pTHS112
215	MB_10B	CTTACTTAGGTACCCGGGGACCGCCAAACAATAGGGTC	*slr1301* for pTHS112
216	MB_12A	TCTAGAGGATCCCCGGGTAATGCTCTATCTGGCTGAAATTAAGAAAC	*slr1301* for pTHS114
217	MB_12B	TCGATGAATTCGAGCTCGGACCGCCAAACAATAGGGTC	*slr1301* for pTHS114
218	MB_13A	TGCCTGCAGGTCGACTCTAATGACAAGTCAAAATTTTGTTTCTGATCAAG	*slr7083* for pTHS99 and pTHS101
219	MB_13B	TCGGTACCCGGGGATCCTCTGGTAAATAAGGGGGAGTGGGAC	*slr7083* for pTHS99 and pTHS101
220	MB_14A	AGGGTCGACTCTAGAGGATATGACAAGTCAAAATTTTGTTTCTGATCAAG	*slr7083* for pTHS100
221	MB_14B	CTTACTTAGGTACCCGGGGTGGTAAATAAGGGGGAGTGGGAC	*slr7083* for pTHS100
222	MB_16A	TCTAGAGGATCCCCGGGTAATGACAAGTCAAAATTTTGTTTCTGATCAAG	*slr7083* for pTHS102
223	MB_16B	TCGATGAATTCGAGCTCGGTGGTAAATAAGGGGGAGTGGGAC	*slr7083* for pTHS102
224	MB_33A	TGCCTGCAGGTCGACTCTAATGCTCTATCTGGCTGAAGTCG	*hmpF*$_{Syc}$ for pTHS115 and pTHS117
225	MB_33B	TCGGTACCCGGGGATCCTCGGCTGCAATCAGTTGATGACT	*hmpF*$_{Syc}$ for pTHS115 and pTHS117
226	MB_34A	AGGGTCGACTCTAGAGGATATGCTCTATCTGGCTGAAGTCG	*hmpF*$_{Syc}$ for pTHS116
227	MB_34B	CTTACTTAGGTACCCGGGGGGCTGCAATCAGTTGATGACT	*hmpF*$_{Syc}$ for pTHS116
228	MB_36A	TCTAGAGGATCCCCGGGTAATGCTCTATCTGGCTGAAGTCG	*hmpF*$_{Syc}$ for pTHS118
229	MB_36B	TCGATGAATTCGAGCTCGGGGCTGCAATCAGTTGATGACT	*hmpF*$_{Syc}$ for pTHS118
230	MB_45A	TGCCTGCAGGTCGACTCTAATGAACTACGCTCTTACCCAAG	*syc2039* for pTHS103 and pTHS105
231	MB_45B	TCGGTACCCGGGGATCCTCAGACCCTAACCAGCGGC	*syc2039* for pTHS103 and pTHS105
232	MB_46A	AGGGTCGACTCTAGAGGATATGAACTACGCTCTTACCCAAG	*syc2039* for pTHS104
233	MB_46B	CTTACTTAGGTACCCGGGGAGACCCTAACCAGCGGC	*syc2039* for pTHS104
234	MB_48A	TCTAGAGGATCCCCGGGTAATGAACTACGCTCTTACCCAAG	*syc2039* for pTHS106

235	MB_48B	TCGATGAATTCGAGCTCGGAGACCCTAA CCAGCGGC	*syc2039* for pTHS106
236	708_Seq_A	CCAACAAACTACCTACCACCAGTC	Verification of Δ*hmpF*$_{Syn}$
237	708_Seq_B	CCGTAGGGATGCCTGATAAACC	Verification of Δ*hmpF*$_{Syn}$
238	Syc879_Seq_A	CATCAGGAATGGATGCAGGAGG	Verification of Δ*hmpF*$_{Syc}$
239	Syc879_Seq_B	GGCCGCTAATCACTTTCAGTG	Verification of Δ*hmpF*$_{Syc}$

**Plasmids**	**Description**	**Resistance**	**Reference**
pJET1.2/ blunt	*E. coli* subcloning vector	Amp	Thermo Fischer Scientific
pMAL-c2x	Bacterial vector for expressing N-terminal MBP-tagged proteins in *E. coli* with a Factor Xa cleavage site	Amp	A gift from Axel Scheidig (University of Kiel)
pet21a(+)	Bacterial vector for expressing C-terminal 6His-tagged proteins in *E. coli*	Amp	Novagen
pRL25C	Shuttle cosmid vector for cyanobacteria and *E. coli*	Km, Nm	[8]
pRL623	Methylation plasmid	Cm	[8]
pRL443	Conjugation plasmid	Amp	[8]
pRL271	*sacB* containing plasmid to select for double homologous recombination in *Anabaena*	Cm	[9]
pRL278	*sacB* containing plasmid to select for double homologous recombination in *Anabaena*	Km, Nm	[9]
pSL2680	Cpf1-mediated CRISPR editing plasmid	Km, Nm	[10]
pRL25c-CRISPR	Functional CRISPR cassette from pSL2680 transferred into EcoRI and BamHI digested pRL25c by GIBSON assembly	Km, Nm	This work
pSM2-Pcpc560ter	pMD18-T derivate for insertion into *pta*, containing P_{cpc560}::*ter*::T_{rbcL} expression cassette	Km, Amp	A gift from Yin Li (Chinese Academy of Science), [11]
pIGA	Cyanobacterial vector for insertion into neutral locus (RS1 and RS2) of *slr0168* in *Synechocystis*	Amp, Km	A gift from Martin Hagemann (University Rostock), [12]
pRL153-GFP	Mobilizable broad host range vector, P_{trc}-*gfp*	Km, Nm	[13]
pKNT25	P_{lac}::-*T25*	Km	Euromedex
pKT25	P_{lac}::*T25*-	Km	Euromedex

pUT18	P_{lac}::*-T18*	Amp	Euromedex
pUT18C	P_{lac}::*T18-*	Amp	Euromedex
pKT25-zip	pKT25; P_{lac}::*T25-zip*	Km	Euromedex
pUT18C-zip	pUT18C, P_{lac}::*T18-zip*	Amp	Euromedex
pAM2991	Cyanobacterial vector for expression of proteins under the control of P_{trc} that inserts into the NS1 site of *Synechococcus*	Sm, Sp	A gift from Susan Golden (Addgene plasmid # 40248)
pTHS1	pRL25C, P_{petE}::*alr4504-gfp*	Km, Nm	This study
pTHS33	pKNT25, P_{lac}::*sepJ-T25*	Km, Nm	This study
pTHS34	pKT25, P_{lac}::*T25-sepJ*	Km, Nm	This study
pTHS35	pUT18, P_{lac}::*sepJ-T18*	Amp	This study
pTHS36	pUT18C, P_{lac}::*T18-sepJ*	Amp	This study
pTHS37	pKNT25, P_{lac}::*ftsZ-T25*	Km, Nm	This study
pTHS38	pKT25, P_{lac}::*T25-ftsZ*	Km, Nm	This study
pTHS39	pUT18, P_{lac}::*ftsZ-T18*	Amp	This study
pTHS40	pUT18C, P_{lac}::*T18-ftsZ*	Amp	This study
pTHS41	pKNT25, P_{lac}::*mreB-T25*	Km, Nm	This study
pTHS42	pKT25, P_{lac}::*T25-mreB*	Km, Nm	This study
pTHS43	pUT18, P_{lac}::*mreB-T18*	Amp	This study
pTHS44	pUT18C, P_{lac}::*T18-mreB*	Amp	This study
pTHS60	pIGA, P_{cpc560}::*fm7001-gfp*::T_{rbcL}	Amp, Km	This study
pTHS61	pET21a(+), P_{T7}::*slr7083-his*	Amp	This study
pTHS62	pET21a(+), P_{T7}::*syc2039-his*	Amp	This study
pTHS63	pET21a(+), P_{T7}::*fm7001-his*	Amp	This study
pTHS64	pET21a(+), P_{T7}::*all4981-his*	Amp	This study
pTHS65	pET21a(+), P_{T7}:: *hmpF*$_{Syn}$*-his*	Amp	This study
pTHS66	pET21a(+), P_{T7}::*hmpF*$_{Syc}$*-his*	Amp	This study
pTHS67	pET21a(+); P_{T7}::*hmpF*$_{Syn}$*-gfp*	Amp	This study
pTHS68	pET21a(+), P_{T7}::*hmpF*$_{Syc}$*-gfp*	Amp	This study
pTHS69	pET21a(+), P_{T7}::s*lr7083-gfp*	Amp	This study
pTHS70	pET21a(+), P_{T7}::*fm7001-gfp*	Amp	This study
pTHS71	pET21a(+), P_{T7}::*syc2039-gfp*	Amp	This study
pTHS72	pET21a(+), P_{T7}::*all4981-gfp*	Amp	This study

pTHS73	pMAL-c2x; P_{tac}::*mbp-fm7001-his*	Amp	This study
pTHS74	pAM2991, P_{trc}::*hmpF$_{Syc}$-gfp*	Sm, Sp	This study
pTHS75	pAM2991, P_{trc}::*syc2039-gfp-his*	Sm, Sp	This study
pTHS76	pIGA, P_{cpc560}::*yfp-fm7001*::T_{rbcL}	Amp, Km	This study
pTHS77	pIGA, P_{cpc560}::*slr7083-gfp*::T_{rbcL}	Amp, Km	This study
pTHS78	pIGA, P_{cpc560}::*yfp-all4981*::T_{rbcL}	Amp, Km	This study
pTHS79	pIGA, P_{cpc560}::*all4981-gfp*::T_{rbcL}	Amp, Km	This study
pTHS80	pIGA, P_{cpc560}::*syc2039-gfp*::T_{rbcL}	Amp, Km	This study
pTHS81	pIGA, P_{cpc560}::*hmpF$_{Syc}$-gfp*::T_{rbcL}	Amp, Km	This study
pTHS82	pRL153, P_{trc}::*hmpF$_{Syn}$-yfp*	Km, Nm	This study
pTHS83	pRL25C, P_{petE}::*fm7001-gfp*	Km, Nm	This study
pTHS84	pRL25C, P_{petE}::*yfp-fm7001*	Km, Nm	This study
pTHS85	pRL25C, P_{petE}::*yfp-slr7083*	Km, Nm	This study
pTHS86	pRL25C, P_{petE}::*slr7083-gfp*	Km, Nm	This study
pTHS87	pRL25C, P_{petE}::*syc2039-gfp*	Km, Nm	This study
pTHS88	pRL25C, P_{petE}::*yfp-all4981*	Km, Nm	This study
pTHS89	pRL25C, P_{petE}::*all4981-gfp*	Km, Nm	This study
pTHS90	pRL25C, P_{petE}-*creS-gfp*	Km, Nm	This study
pTHS91	pRL25C, P_{petE}::*slr1303-gfp*	Km, Nm	This study
pTHS92	pRL25C, P_{petE}::*yfp-slr1303*	Km, Nm	This study
pTHS93	pRL25C, P_{petE}::*hmpF$_{Syc}$-gfp*	Km, Nm	This study
pTHS94	pRL25C, $P_{all4982}$::*all4982-ecfp*	Km, Nm	This study
pTHS95	pKNT25, P_{lac}::*fm7001-T25*	Km, Nm	This study
pTHS96	pKT25, P_{lac}::*T25-fm7001*	Km, Nm	This study
pTHS97	pUT18, P_{lac}::*fm7001-T18*	Amp	This study
pTHS98	pUT18C, P_{lac}::*T18-fm7001*	Amp	This study
pTHS99	pKNT25, P_{lac}::*slr7083-T25*	Km, Nm	This study
pTHS100	pKT25, P_{lac}::*T25-slr7083*	Km, Nm	This study
pTHS101	pUT18, P_{lac}::*slr7083-T18*	Amp	This study
pTHS102	pUT18C, P_{lac}::*T18-slr7083*	Amp	This study
pTHS103	pKNT25, P_{lac}::*syc2039-T25*	Km, Nm	This study
pTHS104	pKT25, P_{lac}::*T25-syc2039*	Km, Nm	This study

pTHS105	pUT18, P_{lac}::*syc2039-T18*	Amp	This study
pTHS106	pUT18C, P_{lac}::*T18-syc2039*	Amp	This study
pTHS107	pKNT25, P_{lac}::*all4981-T25*	Km, Nm	This study
pTHS108	pKT25, P_{lac}::*T25-all4981*	Km, Nm	This study
pTHS109	pUT18, P_{lac}::*all4981-T18*	Amp	This study
pTHS110	pUT18C, P_{lac}::*T18-all4981*	Amp	This study
pTHS111	pKNT25, P_{lac}::*hmpF*$_{Syn}$*-T25*	Km, Nm	This study
pTHS112	pKT25, P_{lac}:: *T25-hmpF*$_{Syn}$	Km, Nm	This study
pTHS113	pUT18, P_{lac}::*hmpF*$_{Syn}$*-T18*	Amp	This study
pTHS114	pUT18C, P_{lac}::*T18-hmpF*$_{Syn}$	Amp	This study
pTHS115	pKNT25, P_{lac}::*hmpF*$_{Syc}$*-T25*	Km, Nm	This study
pTHS116	pKT25, P_{lac}::*T25-hmpF*$_{Syc}$	Km, Nm	This study
pTHS117	pUT18, P_{lac}::*hmpF*$_{Syc}$*-T18*	Amp	This study
pTHS118	pUT18C, P_{lac}::*T18-hmpF*$_{Syc}$	Amp	This study
pTHS119	pJET1.2/blunt with ~1000 bp upstream and downstream of *syc2039* flanking *nptII*	Amp	This study
pTHS120	Circularized pUC ori with 1000 bp upstream and downstream of *slr7083* flanking *nptII* assembled by GIBSON assembly	Km, Nm	This study
pTHS121	pSL2680 with *fm7001* gRNA and homologous repair templates 1000 bp upstream and downstream of *fm7001*	Km, Nm	This study
pTHS122	pRL25C containing *cpf1*, *lacZα* and pre-crRNA array with tandem spacer-repeat sequences from *Francisella novicida*	Km, Nm	This study
pTHS123	pTHS122 with *fm7001* gRNA and homologous repair templates 1000 bp upstream and downstream of *fm7001*	Km, Nm	This study
pTHS124	pSL2680 with *all4981* gRNA and homologous repair templates 1000 bp upstream and downstream of *all4981*	Km, Nm	This study
pTHS125	pTHS122 with *all4981* gRNA and homologous repair templates 1000 bp upstream and downstream of *all4981*	Km, Nm	This study
pTHS126	pRL271 containing 1000 bp upstream and downstream of *fm7001* flanking *nptII*	Km, Nm, Cm	This study
pTHS127	pRL278 containing 2000 bp upstream and downstream of *fm7001* flanking CS.3	Km, Nm, Sm, Sp	This study
pTHS128	pRL278 containing 2000 bp upstream of *fm7001* and the first 398 bp of *fm7001*	Km, Nm	This study

pTHS129	pRL278 containing 1000 bp upstream and downstream of *all4981* flanking CS.3	Km, Nm, Sm, Sp	This study
pTHS130	pRL278 containing 151 bp upstream of *all4981* and the first 449 bp of *all4981*	Km, Nm	This study
pTHS131	pJET1.2/blunt with ~1000 bp upstream and downstream of *slr1303* flanking CS.3 inserted by GIBSON assembly	Amp	This study
pTHS132	Circularized pUC ori with 1000 bp upstream and downstream of *slr1301* ($hmpF_{Syn}$) flanking *nptII* assembled by GIBSON assembly	Km, Nm	This study
pTHS133	pJET1.2/blunt with ~1000 bp upstream and downstream of *syc1139* ($hmpF_{Syc}$) flanking *nptII* inserted by GIBSON assembly	Amp	This study

Restriction sites or overlapping sites are underlined.

Sm: streptomycin resistance; Sp: spectinomycin resistance; Amp: ampicillin resistance, Km: kanamycin resistance, Nm: neomycin resistance; Cm: chloramphenicol resistance.

- 1) The eYFP is C-terminally followed by a myc-tag, which is then followed by a heptapeptide of glycine and serine. Abbreviated: *yfp*.
- 2) Modified *gfpmut3.1* in which the internal NdeI site was removed by replacing CAT by the synonymous CAC codon. The GFP is N-terminally preceded by 12 alanine and serine residues[14]. Abbreviated: *gfp*.

Supplementary references

1. Corpet, F. Multiple sequence alignment with hierarchical clustering. *Nucleic Acids Res.* **16**, 10881–90 (1988).
2. Yang, J. & Zhang, Y. I-TASSER server: New development for protein structure and function predictions. *Nucleic Acids Res.* **43**, W174–W181 (2015).
3. Zhang, Y. I-TASSER: Fully automated protein structure prediction in CASP8. *Proteins Struct. Funct. Bioinforma.* **77**, 100–113 (2009).
4. Boyer, H. & Roulland-Dessoix, D. A complementation analysis of the restrcition and modification of DNA in Escherichia coli. *J. Mol. Biol.* **41**, 459–472 (1969).
5. Meselson, M. & Yuan, R. DNA restriction enzyme from E. coli. *Nature* **217**, 1110–4 (1968).
6. Grant, S. G., Jessee, J., Bloom, F. R. & Hanahan, D. Differential plasmid rescue from transgenic mouse DNAs into Escherichia coli methylation-restriction mutants. *Proc. Natl. Acad. Sci.* **87**, 4645–4649 (1990).

7. Studier, F. W. & Moffatt, B. A. Use of bacteriophage T7 RNA polymerase to direct selective high-level expression of cloned genes. *J. Mol. Biol.* **189**, 113–130 (1986).

8. Wolk, C. P. *et al.* Isolation and complementation of mutants of Anabaena sp. strain PCC 7120 unable to grow aerobically on dinitrogen. *J. Bacteriol.* **170**, 1239–1244 (1988).

9. Cai, Y. & Wolk, C. P. Use of a conditionally lethal gene in Anabaena sp. strain PCC 7120 to select for double recombinats and to entrap insertion sequnces. *J. Bacteriol.* **172**, 3138–3145 (1990).

10. Ungerer, J. & Pakrasi, H. B. Cpf1 Is A Versatile Tool for CRISPR Genome Editing Across Diverse Species of Cyanobacteria. *Sci. Rep.* **6**, 1–9 (2016).

11. Zhou, J. *et al.* Discovery of a super-strong promoter enables efficient production of heterologous proteins in cyanobacteria. *Sci. Rep.* **4**, 1–6 (2014).

12. Kunert, A., Hagemann, M. & Erdmann, N. Construction of promoter probe vectors for Synechocystis sp. PCC 6803 using the light-emitting reporter systems Gfp and LuxAB. *J. Microbiol. Methods* **41**, 185–194 (2000).

13. Tolonen, A. C., Liszt, G. B. & Hess, W. R. Genetic manipulation of Prochlorococcus strain MIT9313: green fluorescent protein expression from an RSF1010 plasmid and Tn5 transposition. *Appl. Environ. Microbiol.* **72**, 7607–7613 (2006).

14. Stucken, K., Ilhan, J., Roettger, M., Dagan, T. & Martin, W. F. Transformation and conjugal transfer of foreign genes into the filamentous multicellular cyanobacteria (subsection V) Fischerella and Chlorogloeopsis. *Curr. Microbiol.* **65**, 552–560 (2012).
